# From bead to flask: Synthesis of a complex β-amido-amide for probe-development studies

**DOI:** 10.3762/bjoc.9.31

**Published:** 2013-02-06

**Authors:** Kevin S Martin, Cristian Soldi, Kellan N Candee, Hiromi I Wettersten, Robert H Weiss, Jared T Shaw

**Affiliations:** 1 Department of Chemistry, University of California, Davis, CA 95616, USA; 2 Comparative Pathology Graduate Group, University of California, Davis, CA 95616, USA; 3 Division of Nephrology, Dept. of Internal Medicine, University of California, Davis, Medical Center, Sacramento, CA 95817, USA; 4 UC Davis Comprehensive Cancer Center, 2279 45th Street Sacramento, CA 95817, USA

**Keywords:** β-amino acid, benzimidazole, multicomponent reaction

## Abstract

A concise synthesis of benzimidazole-substituted β-amido-amide LLW62 is presented. The original synthesis of compounds related to LLW62 was developed on Rink resin as part of a “one-bead, one-compound” combinatorial approach for on-bead screening purposes. The current synthesis is carried out in solution and is amenable to scale-up for follow-up studies on LLW62 and investigations of related structures. The key step involves the use of a β-amino acid-forming three-component reaction (3CR), the scope of which defines its role in the synthetic strategy.

## Introduction

**Figure 1:**
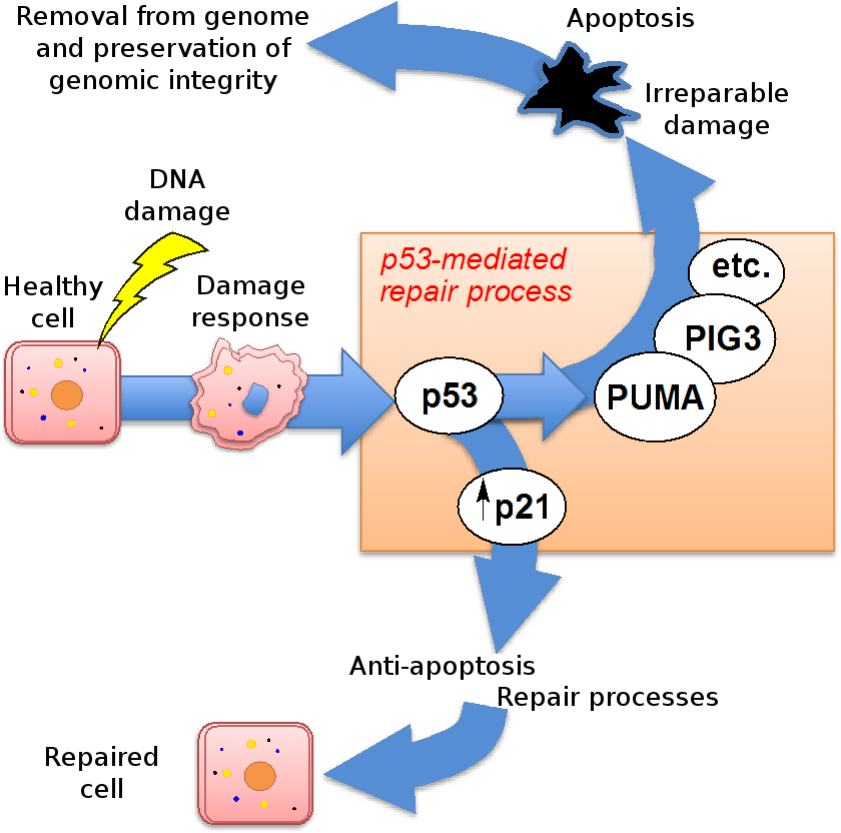
p21 determines the fate of DNA-damaged [13] cells.

**Figure 2:**
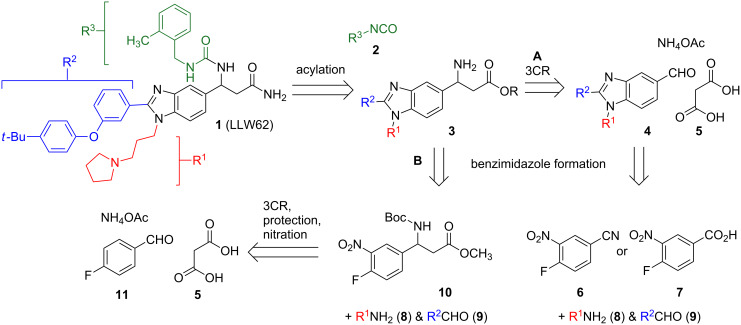
Retrosynthetic analysis of LLW62 (**1**) from acid **7** (A) or aldehyde **11** (B).

Library syntheses and high-throughput screening can often be combined to enable the discovery of new small-molecule probes that modulate biological phenomena [1]. Although the use of solid-phase, split-pool combinatorial synthesis for the preparation of solutions of small-molecule libraries has declined, the use of these compounds for on-bead screening has resulted in recent screening innovations [1–2]. The Lam and Kurth groups have published several “one-bead, one-compound” (OBOC) library syntheses of heterocyclic structures for a variety of screening endeavors [3–12]. Some of these compounds were identified as inhibitors of p21, which is a protein that modulates the activity of cyclin kinases [13–15]. One function of p21 is that it acts downstream of p53 to repair DNA-damaged cells and may function to convey anti-apoptotic activity to cancer cells (Figure 1) [13]. As such, an inhibitor of p21 could sensitize malignant cells to DNA-damaging chemical and radiation therapy by subverting this p21-mediated DNA repair process [14–17]. In this study, we developed a synthesis of LLW62 (**1**, Figure 2), which is a complex benzimidazole-substituted β-amido-amide similar in structure to inhibitors of p21 that were reported previously to support studies of this compound as a biological probe [14–15].

The synthesis of **1** emanates from a one-pot, three-component reaction (3CR) of an arylaldehyde, malonic acid (**5**), and ammonium acetate, which assembles the β-amino acid core (Figure 2) [14–15,18]. In the reported synthesis of **1**, a protected β-amino acid core was attached to Rink-amide resin and carried through to **1** by a series of elaboration and tagging steps [14–15]. Synthetic intermediates in this route were not characterized, and **1** was ultimately purified by high-performance liquid chromatography and partially characterized by matrix-assisted laser desorption/ionization mass spectrometry [14]. In the current synthesis, we set out to develop a concise and scalable solution-phase route to **1** and provide characterization data for **1** and all intermediate compounds.

In our retrosynthetic analysis, we envisioned **1** coming from acylation of benzimidazole **3** with isocyanate **2** (Figure 2). We initially sought to avoid nitration, protection and deprotection steps and access this intermediate by performing a late-stage 3CR with benzimidazole **4**, which would be available from nitrile **6** or acid **7** (Figure 2, A). Although synthesis of **4** proceeded without difficulty from acid **7**, this route was unsuccessful at a late stage for a reason that we describe below. We next envisioned benzimidazole **3** emanating from β-amino ester **10,** which could be accessed in a few steps starting with an early stage 3CR of aldehyde **11**, malonic acid (**5**), and ammonium acetate (Figure 2, B). Gratifyingly, **10** was converted to the requisite benzimidizole **3** in three steps and carried through to **1**.

## Results and Discussion

**Scheme 1:**
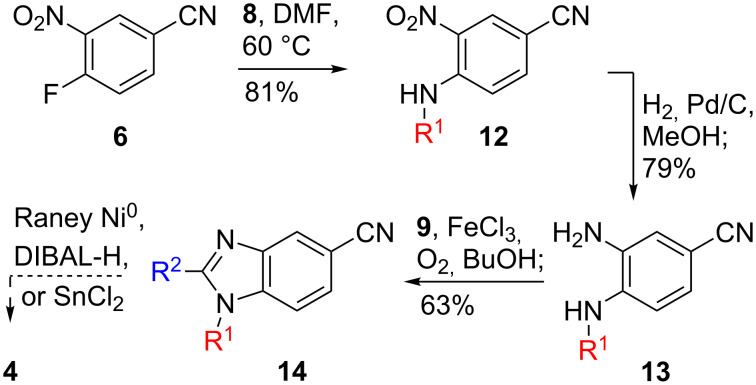
Attempted synthesis of aldehyde **4** from nitrile **6**.

Our initial target was benzimidazole **4**, which we envisioned originating from nitrile **6** or acid **7**, each of which is commercially available (Figure 2). We first attempted to synthesize **4** from **6**, which would lead to the shortest possible synthesis of **1**. Nitrile **6** was treated with *N*-(3-aminopropyl)pyrrolidine (**8**) to produce aniline **12** in 81% yield (Scheme 1) [19]. This compound was reduced to aniline **13** in 79% yield and converted to the benzimidizole **14** in 63% yield with aldehyde **9** under oxidative conditions. The resultant nitrile proved to be extremely insoluble and difficult to handle. Several reduction conditions were attempted to produce benzimidazole **4** with no success. In addition, attempts to use the nitrile in a Blaise-type reaction or similar nucleophilic addition were also unsuccessful (not shown). Although nitrile **6** would have provided the shortest, most direct entry into the requisite β-amino core structure, we turned our attention to another route to **4**.

**Scheme 2:**
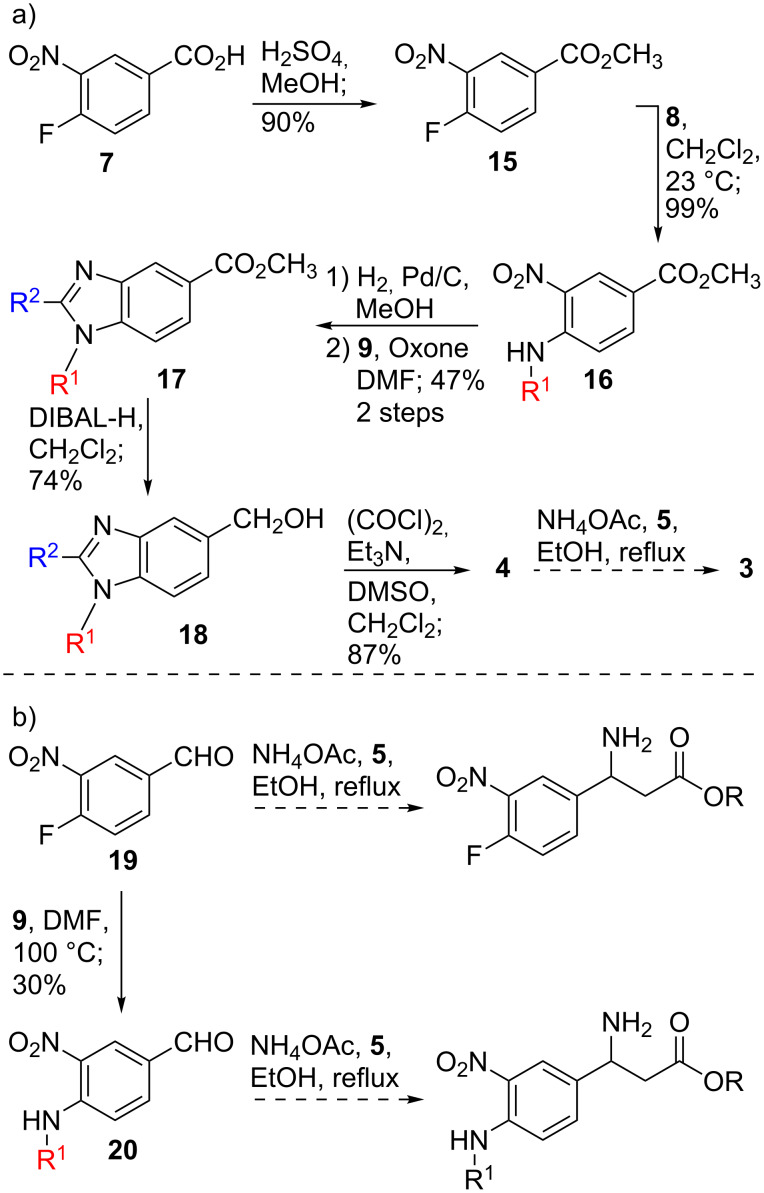
(A) Synthesis of **4** from acid **7** and (B) attempted β-amino acid-forming 3CRs.

Acid **7** was immediately more promising as a starting material for benzimidazole **4**. Commercially available **7** was converted to methyl ester **15** in 90% yield, due to its ease of handling (Scheme 2) [20]. Next, S_N_Ar displacement of the fluoride of **15** by *N*-(3-aminopropyl)pyrrolidine (**8**) proceeded in high yield, 99%, to give aniline **16** [20]. Reduction of the nitro group was nearly quantitative and subsequent benzimidazole formation with Oxone furnished benzimidazole **17** in 47% yield over two steps [20–21]. The ester of **17** was smoothly reduced to the alcohol **18**, in 74% yield, and immediately oxidized to the aldehyde **4**, in 87% yield. Unfortunately, **4** produced none of the desired β-amino acid **3** under several different variants of the 3CR with malonic acid (**5**) and ammonium acetate. Tan and Weaver demonstrated previously that the β-amino acid forming 3CR works best for electron-rich aldehydes and poorly for electron-deficient aldehydes [18]. Thus, we suspected that aldehyde **4** may be too electron poor for the 3CR to work efficiently. Our suspicions were supported by attempting 3CRs on aldehydes **19** and **20**, each of which has a single nitro group, and neither was successful in this transformation.

**Scheme 3:**
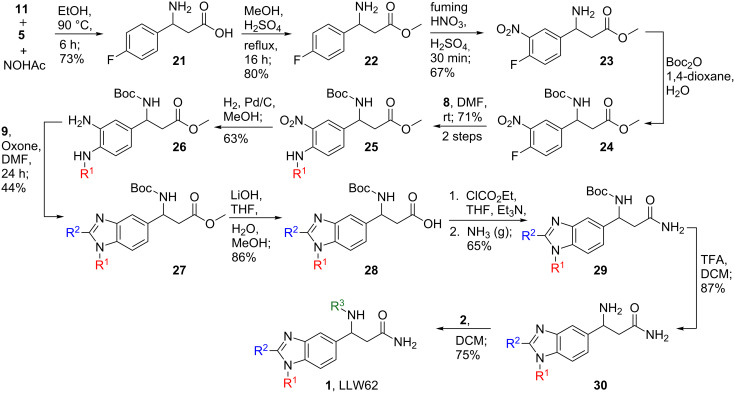
Synthesis of LLW62 by using an early stage 3CR.

An early-stage 3CR enabled the use of the least electron-poor aldehyde in this key step (Scheme 3). Heating of 4-fluorobenzaldehyde (**11**), malonic acid and ammonium acetate under reflux proceeded smoothly, as previously described, to furnish β-amino acid **21** in 73% yield [18]. Methylation of **21** (80%) followed by nitration of **22** (67%), boc protection of **23** and S_N_Ar displacement of the fluoride in **24** with amine **8** (71% over two steps), and finally reduction of the nitro group of **25** (63%) provided aniline **26** as our key intermediate for forming the benzimidazole core of **1**. We next attempted to produce **27** under the higher yielding oxidative conditions described for the formation of nitrile containing benzimidazole **14**. The yield for this reaction was significantly lower, less than 50%, compared to the reaction to produce **14**, and we observed some transesterification of the methyl ester with butanol to produce a mixture of **27** and the butyl ester of **27** as the major products (not shown). We thus turned to using Oxone, and benzimidazole formation proceeded in acceptable yield (44%) from aniline **26** to furnish **27.** Benzimidazole **27** was then saponified under basic conditions to give acid **28** (86%) [21]. Installation of the primary amide of **1** was then achieved in a single pot by treatment of **28** with ethyl chloroformate to make the mixed anhydride followed by displacement with ammonia gas to produce **29** in 65% yield [22]. Final Boc deprotection of **29** with TFA (87%) and subsequent acylation of the free amine of **30** with isocyanate **2** (75%) provided the desired compound **1** in 11 total steps and 3% overall yield.

## Conclusion

We have completed a solution-phase synthesis of **1** and thus provided a common route to related compounds that may emerge from future on-bead screening experiments. The key step was the 3CR to form the β-amino acid core structure. Although the electronic requirements of this reaction limit it to electron-rich, or at least not excessively electron poor, aromatic aldehydes, application of this transformation early in the synthesis ultimately proved successful. Although this route is not suitable for large-scale production of **1**, multigram quantities of this compound and benzimidazoles of comparable complexity are easily accessible for early stage studies of these compounds in vitro and in vivo using model organisms.

## Supporting Information

File 1 Experimental procedures and compound characterization.
