# Supplementary material for: From bead to flask: Synthesis of a complex β-amido-amide for probe-development studies
Source: Beilstein J Org Chem. 2013 Feb 6;9:260–4. doi: 10.3762/bjoc.9.31 (PMC3566855; doi:10.3762/bjoc.9.31)
Supplement: File 1 — Experimental procedures and compound characterization. [file Beilstein_J_Org_Chem-09-260-s001.pdf]

Supporting Information

for

From bead to flask: Synthesis of a complex β-amido-amide for probe-development studies

Kevin S. Martin[1,2], Cristian Soldi[1], Kellan N. Candee[1], Hiromi I. Wettersten[2,3], Robert H. Weiss[2,3,4] and Jared T. Shaw[1,2,4]*

Address: [1]Department of Chemistry, University of California, Davis, CA 95616, USA, [2]Comparative Pathology Graduate Group, University of California, Davis, CA 95616, USA, [3]Division of Nephrology, Dept. of Internal Medicine, University of California, Davis, Medical Center, Sacramento, CA 95817, USA and [4]UC Davis Comprehensive Cancer Center, 2279 45th Street Sacramento, CA 95817, USA
*Corresponding author
Email: Jared T. Shaw – jtshaw@ucdavis.edu

Experimental procedures and compound characterization

Table of Contents Page

1. Materials and instrumentation S1
2. Preparation of aldehyde **4** from nitrile **6** S3
3. Preparation of aldehyde **4** from acid **7** S5
4. Synthesis of **1** from the β-amino-acid-forming three-component reaction S10
5. References S19
6. ^{1}H and ^{13}C NMR spectra S19

1. Materials and instrumentation: Unless otherwise specified, all commercially available reagents were used as received. All reactions using dried solvents were carried out under an atmosphere of argon in flame-dried glassware with magnetic stirring. Dry solvent was dispensed from a solvent purification system that passes solvent through two columns of dry neutral alumina.

^{1}H NMR spectra and proton-decoupled ^{13}C NMR spectra were obtained on a 300, 400 or 600 MHz Varian NMR spectrometer. Chemical shifts (δ) are reported in parts per million (ppm) relative to TMS (s, δ 0). Multiplicities are given as: s (singlet), d (doublet), t (triplet), dd (doublet of doublets), m (multiplet), br m (broad multiplet), br s (broad singlet). ^{13}C NMR chemical shifts are reported relative to $CDCl_3$ (t, δ 77.4) unless otherwise noted. High-resolution mass spectra were recorded on positive ESI mode in methanol or acetonitrile. Melting points were taken on an EZ-melting apparatus and were uncorrected. Infrared spectra were taken on a Bruker Tensor 27 spectrometer. Chromatographic purifications were performed by flash chromatography with silica gel (Silicycle, 40–63 μm) packed in glass columns. The eluting solvent for the purification of each compound was determined by thin-layer chromatography (TLC) on glass plates coated with EMD silica gel 50 F_{254} and visualized by ultraviolet light.

Abbreviations for frequently used chemicals will be seen as follows: dichloromethane (DCM), ethanol (EtOH), ethyl acetate (EtOAc), methanol (MeOH), tetrahydrofuran (THF), triethylamine (TEA).

2. Preparation of aldehyde 4 from nitrile 6

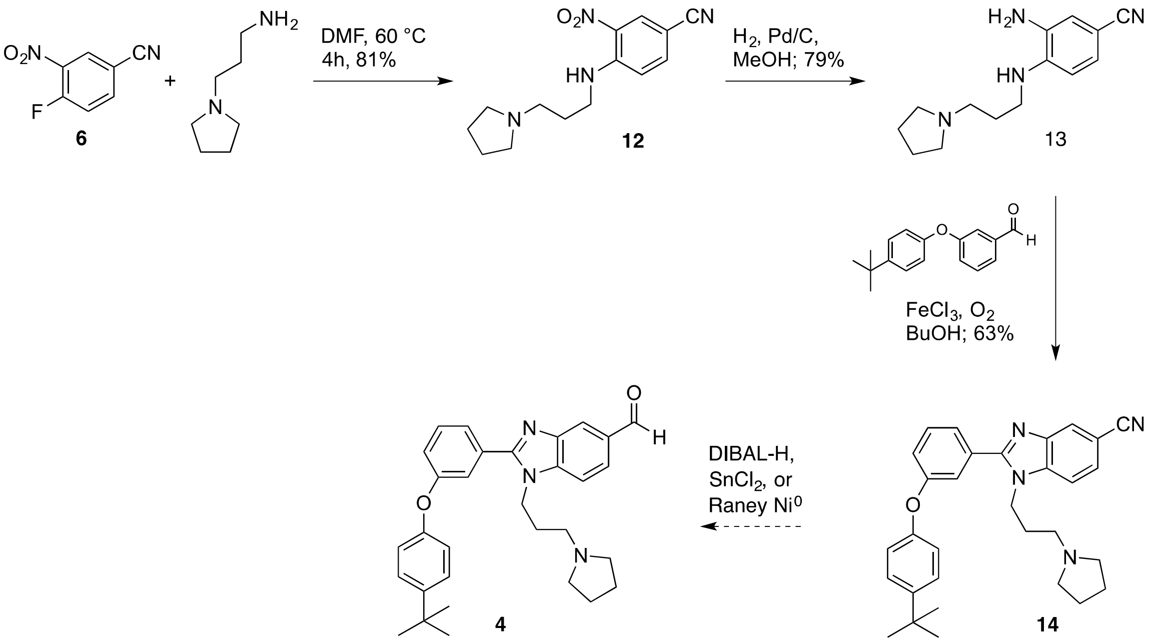

Compound 12

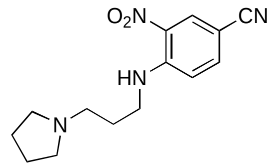

To a solution of fluoro compound **6** (1.50 g, 8.2 mmol) in THF (3 mL) at 0 °C, was added the *N*-(pyrrolidinyl)propylamine (**8**, 1.15 g, 9.4 mmol). The reaction mixture was heated to 60 °C and stirred for 4 hours. The crude mixture was cooled to room temperature and water was added. The yellow solid was filtered using a fritted funnel and was washed with MeOH. The yellow solid was dried under high vacuum to yield 1.83 g (81%) of product. ^{1}H NMR (600 MHz, $CDCl_3$) δ 9.14 (s, 1H), 8.50 (d, J = 2.1 Hz, 1H), 7.57 (dd, J = 9.0, 2.1 Hz, 1H), 6.94 (d, J = 9.0 Hz, 1H), 3.45 (td, J = 6.5, 4.9 Hz, 2H), 2.65 (t, J = 6.3 Hz, 2H), 2.55 (m, 4H), 1.93 (p, J = 6.4 Hz, 2H), 1.84 (m, 4H); ^{13}C NMR (150 MHz, $CDCl_3$) δ 147.2, 137.3, 132.2, 131.4, 118.2, 114.8, 97.4, 54.5, 54.3, 43.0, 27.0, 23.5; mp 116.9–117.5 °C; IR (neat): 3209, 2213, 1616 cm^{-1}.

Compound 13

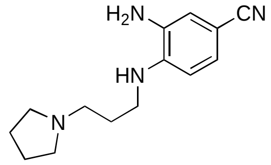

To a round-bottom flask purged with Ar was added EtOH (20 mL) and Pd/C 10% (1.03 g, 0.76 mmol). The nitro compound **12** was added (0.600 g, 2.2 mmol), and the Ar atmosphere was switched to H_2 at 1 atm. The reaction mixture was stirred at room temperature for 2 hours. Then, the crude mixture was filtered through a pad of celite and the residue was washed with EtOH. The solvent was removed under reduced pressure and the product was dried under high vacuum yielding 0.423 g (79%) of product as a white solid. ^{1}H NMR (600 MHz, $CDCl_3$, 50 °C) δ 7.09 (dd, *J* = 8.2, 1.9 Hz, 1H), 6.86 (d, *J* = 1.9 Hz, 1H), 6.51 (d, *J* = 8.2 Hz, 1H), 3.34 (br s, 2H), 3.26 (t, *J* = 6.2 Hz, 2H), 2.67 (t, *J* = 6.2 Hz, 2H), 2.57 (m, 4H), 1.89 (q, *J* = 6.2 Hz, 2H), 1.81 (m, 4H); ^{13}C NMR (150 MHz, $CDCl_3$, 50 °C) δ 142.4, 133.1, 126.2, 120.6, 118.2, 109.4, 98.7, 55.3, 54.1, 43.6, 26.9, 23.5; M.p. 94.0 - 95.2 ^{o}C; IR (film in CH_2Cl_2): 3535, 3369, 2209 cm^{-1}.

Compound 14

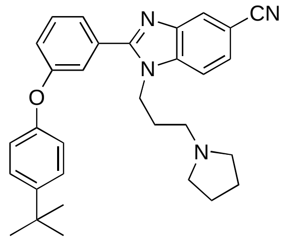

The amino compound **13** (0.100 g, 0.41 mmol) and the aldehyde **9** (0.104 g, 0.41 mmol) were combined in *n*-butanol (1 mL) and heated to 90 °C. The reaction mixture was stirred at 90 °C for 2 hours. Then, the reaction mixture was cooled to room temperature and $FeCl_3$ (0.0055 g, 0.020 mmol) was added. O_2(g) was bubbled into the solution for 5 minutes, then the reaction mixture was heated to 90 °C and stirred overnight. The crude mixture was quenched with water. Hexanes were added and the solid formed was filtered and then dissolved in

DCM, and the organic layer was washed with brine. The organic layer was dried over Na_2SO_4, and the solvent was removed under reduced pressure yielding 0.124 g (63%) of product as a slightly brown solid. ^{1}H NMR (400 MHz, $CDCl_3$, 50 °C) δ 8.10 (br s, 1H), 7.54 (br s, 2H), 7.48 (t, *J* = 7.9 Hz, 1H), 7.42 (dt, *J* = 7.6, 1.3 Hz, 1H), 7.37 (d, *J* = 8.8 Hz, 2H), 7.34 (m, 1H), 7.17 (ddd, *J* = 8.1, 2.5, 1.1 Hz, 1H), 6.99 (d, *J* = 8.8 Hz, 2H), 4.38 (t, *J* = 7.4 Hz, 2H), 2.41 (br s, 6H), 1.95 (br s, 4H), 1.77 (s, 2H), 1.32 (s, 9H); ^{13}C NMR (150 MHz, $CDCl_3$, 50 °C) δ 158.4, 155.7, 154.0, 147.1, 142.7, 138.6, 131.2, 130.5, 127.0, 126.3, 125.1, 123.7, 120.4, 120.0, 119.1, 119.0, 111.4, 105.2, 54.1, 52.7, 43.1, 34.5, 31.6, 29.9, 23.6; mp 142.9–144.7 °C; IR (neat): 2959, 2216 cm^{-1}.

3. Preparation of aldehyde 4 from acid 7

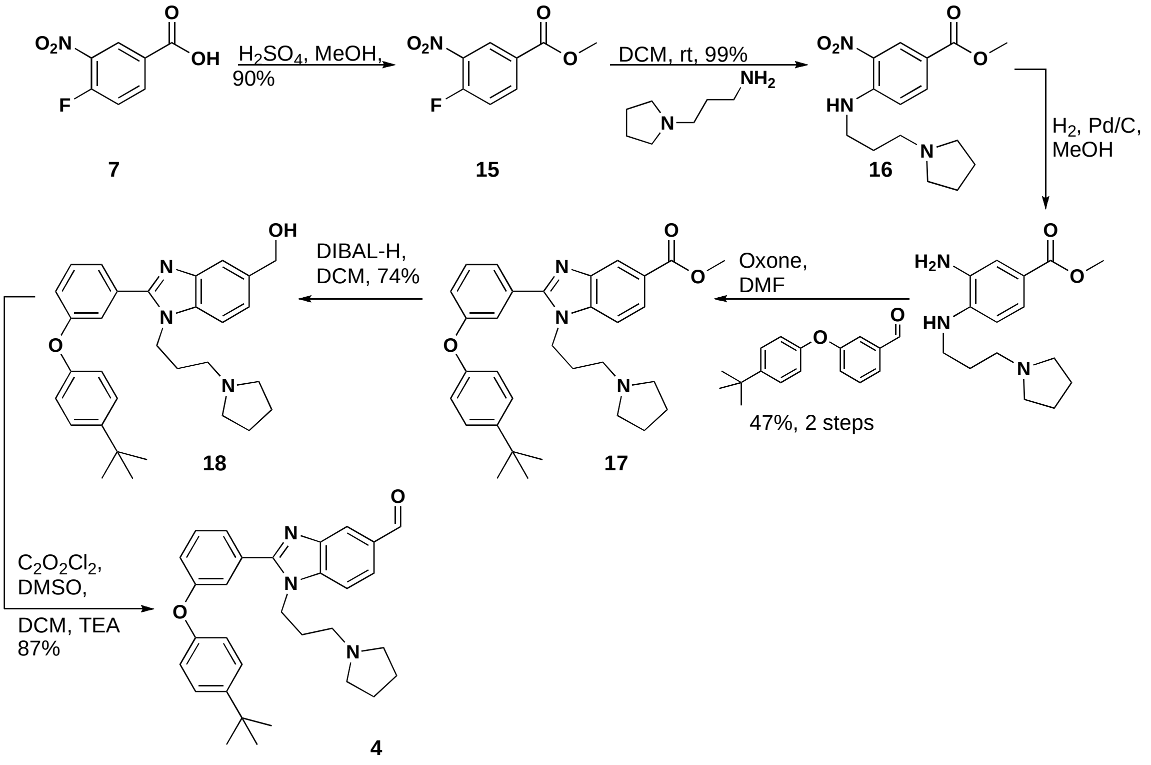

Compound 15

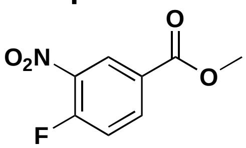

Aldehyde **4** was prepared as described in the literature [1]. Concentrated H_2SO_4 (0.575 mL, 11 mmol) was added to a solution of 4-fluoro-3-nitrobenzoic acid (**7,** 1.00 g**,** 5.4 mmol) in MeOH (25 mL) at room temperature and the mixture was heated under reflux for 6 hours. After cooling to room temperature, EtOAc was added and MeOH was removed under reduced pressure. The resulting mixture was then diluted with water and the aqueous phase was neutralized with 1 M NaOH. The aqueous phase was extracted with DCM (2 × 20 mL), and then the combined organic extracts were dried over Na_2SO_4 and concentrated. Purification by flash chromatography (20 to 80% EtOAc/hexanes) afforded the title compound as a yellow solid (0.709 g, 66%). ^{1}H NMR (400 MHz, $CDCl_3$) δ = 8.73 (dd, J = 7.2, 2.2 Hz, 1H), 8.32 (ddd, J = 8.7, 4.3, 2.2 Hz, 1H), 7.38 (dd, J = 10.2, 8.8 Hz, 1H), 3.97 (s, 1H). ^{1}H NMR matches what was reported in the literature [1].

Compound 16

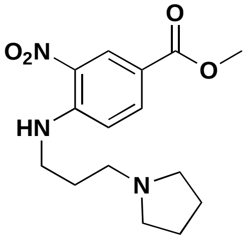

Compound **15** (0.500 g, 2.5 mmol) was added to a flame-dried flask and dissolved in DCM (5 mL). The resulting solution was then added dropwise into a flame-dried flask containing a solution of 1-(3-aminopropyl)pyrrolidine (**8**, 0.380 mL, 2.8 mmol) at room temperature. Triethylamine (0.740 mL, 5.3 mmol) was added to the resulting mixture in four portions over the course of 25 hours. After 25 hours, DCM (20 mL) was added to the reaction followed by water (25 mL).

The aqueous layer was then basified to pH 10 with a 10% NaOH solution (15 mL). The organic layer was then separated and the aqueous layer extracted with DCM (2 × 20 mL). Combined organic extracts were washed with brine and $NaHCO_3$ and dried over Na_2SO_4. Purification by flash chromatography (5 to 10% MeOH/DCM, 0.2% TEA) afforded the title compound as a yellow oil (0.768 g, 99%). ^{1}H NMR (600 MHz, $CDCl_3$) δ 8.80 (d, J = 2.1 Hz, 1H), 8.63-8.61 (m, 1H), 8.03 (dd, J = 9.0, 2.1 Hz, 1H), 6.93 (d, J = 9.0, 1H), 3.89 (s, 3H), 3.60-3.57 (m, 2H), 3.04-3.00 (m, 6H), 2.21-2.16 (m, 2 H), 2.07-2.01 (m, 4H); ^{13}CNMR (151 MHz, $CDCl_3$) δ 165.5, 147.4, 136.3, 131.3, 129.36, 117.21, 113.5, 54.0, 53.4, 52.1, 41.3, 26.1, 23.4; IR (neat): 3379, 2949, 1718, 1621, 1217 cm^{-1}.

Compound 17

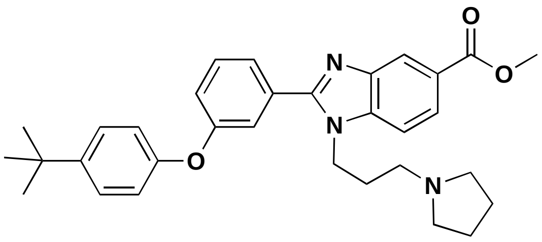

Compound **16** (0.228 g, 0.74 mmol) was added to a flame-dried flask, and the flask was purged with Ar. 10% Pd on activated carbon (0.057 g) was added to the flask, followed by dry MeOH (5 mL), and the flask was further purged with Ar. The Ar needle was removed and a balloon of H_2(g) was added. After briefly purging with H_2(g) to remove all Ar, the reaction was stirred under an H_2(g) atmosphere overnight. After 17 hours, the H_2 balloon was removed and reaction contents were filtered through celite and concentrated under reduced pressure to give a crude mixture of amine compound as a red oil (0.201 g). This crude mixture appeared pure by NMR and was carried on to the following reaction without further purification.

To the crude mix of the amine compound (0.201 g, 0.73 mmol) was added 3-(4-*tert*-butylphenoxy)benzaldehyde (**9**, 0.184 g, 0.73 mmol) followed by DMF (4.5 mL) and H_2O (0.200 mL). Oxone (0.267 g, 0.44 mmol) was added in 4 portions

over the course of 15 minutes and the reaction was stirred overnight. After 17 hours, H_2O (10 mL) was added to the reaction, and the aqueous layer was extracted with DCM (3 × 15 mL). Combined organic extracts were washed with brine, dried over Na_2SO_4, filtered and concentrated to give a brown oil. Flash chromatography (5 to 10% MeOH/DCM, 0.2% TEA) afforded the title compound as a yellow oil (0.177 g, 47% over two steps). ^{1}H NMR (400 MHz, $CDCl_3$) δ 8.51-8.50 (m, 1H), 8.05-8.02 (m, 1H), 7.50-7.43 (m, 3H), 7.38-7.34 (m, 3H), 7.18-7.15 (m, 1H), 7.01-6.98 (m, 2H), 4.37-4.33 (m, 2H), 3.95 (s, 3H), 2.39-2.34 (m, 6H), 1.97-1.89 (m, 2H), 1.75-1.72 (m, 4H), 1.32 (s, 9H); ^{13}CNMR (101 MHz, $CDCl_3$) δ 167.6, 158.1, 154.8, 154.0, 146.8, 142.5, 139.0, 131.6, 130.2, 126.8, 124.6, 124.4, 123.7, 122.3, 119.9, 118.9, 118.9, 109.9, 53.9, 52.8, 52.1, 43.0, 34.4, 31.5, 29.0, 23.4; IR (neat): 2948, 1712, 1592, 1458, 1224 cm^{-1}.

Compound 18

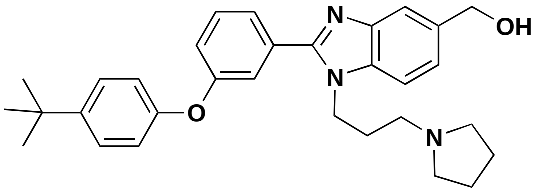

Compound **17** (0.160 g, 0.31 mmol) was added to a flame-dried flask and dissolved in DCM (1.4 mL). After purging the flask with Ar, diisobutylaluminium hydride (1.25 mL, 1.25 mmol, 1 M in hexanes) was added at −78 °C and the reaction was allowed to warm to room temperature overnight. After 17 hours, the reaction was diluted with ether and cooled to 0 °C. Water (0.05 mL) was carefully added to the reaction followed by 15% NaOH solution (0.05 mL). Water (0.125 mL) was added and the mixture was allowed to warm to room temperature while being stirred for 15 minutes. Anhydrous $MgSO_4$ was added and the mixture was stirred for an additional 15 minutes after which time the mixture was filtered and the layers were separated. The aqueous layer was extracted with DCM (2 × 20 mL), and then the organic layers were combined, dried over Na_2SO_4, filtered and concentrated. Flash chromatography (5 to 10% MeOH/DCM, 0.2% TEA) afforded

the title compound **18** as a thin yellow oil (0.113 g, 74%). ^{1}H NMR (600 MHz, $CDCl_3$) δ 7.70 (s, 1H), 7.44-7.41 (m, 2H), 7.37-7.30 (m, 5H), 7.14-7.12 (m, 1H), 7.00-6.98 (m, 2H) 5.28 (s, 1H), 4.73 (s, 2H), 4.27-4.25 (m, 2H) 2.36-2.31 (m, 6H), 1.92-1.87 (m, 2H), 1.73-1.70 (m, 4H), 1.31 (s, 9H); ^{13}CNMR (101 MHz, $CDCl_3$) δ 158.2, 154.4, 153.6, 146.9, 143.1, 136.3, 135.3, 132.3, 130.3, 126.9, 124.0, 122.8, 119.9, 119.3, 119.0, 118.6, 110.4, 65.6, 54.2, 53.2, 43.2, 34.6, 31.7, 29.3, 23.6; IR (neat): 3261, 2960, 2877, 2796, 1578 cm^{-1}.

Compound 4

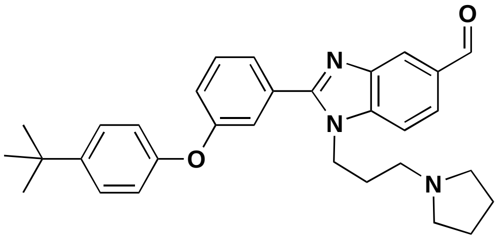

DMSO (0.042 mL, 0.59 mmmol) in DCM (0.150 mL) was added slowly to a solution of oxalyl chloride (0.022 mL, 0.26 mmol) in DCM (0.600 mL) at −78 °C over the course of 5 minutes, and the resulting mixture was stirred at −78 °C for 10 minutes. After 10 minutes, alcohol compound **18** (0.095 g, 0.20 mmol) was dissolved in DCM (1.3 mL) and was added to the mixture at −78 °C over the course of 5 minutes. After 2 hours, TEA (0.190 mL, 1.4 mmol) was added and the reaction was stirred for an additional 2 hours at −78 °C. After 2 hours, H_2O (1.3 mL) and DCM (0.800 mL) were added and the reaction was allowed to warm to room temperature. DCM (10 mL) and water (10 mL) were added to the reaction and the layers were separated. The aqueous layer was extracted with DCM (2 × 10 mL) and the combined organic layers were dried over Na_2SO_4, filtered and concentrated. Flash chromatography (5 to 10% MeOH/DCM, 0.2% TEA) yielded the title compound **4** as a yellow oil (0.082 g, 87%). ^{1}H NMR (600 MHz, $CDCl_3$) δ 10.09 (s, 1H), 8.28 (dd, J = 1.6, 0.7 Hz, 1H), 7.91 (dd, J = 8.5, 1.5 Hz, 1H), 7.58 (d, J = 8.4 Hz, 1H) 7.51-7.48 (m, 2H), 7.45-7.43 (m, 1H), 7.39-7.35 (m, 2H), 7.19-7.17 (m, 1H), 7.01-6.99 (m, 2H), 4.39 (t, J = 7.5 Hz, 2H), 2.46-2.41 (m, 6H), 1.97 (p, J = 7.1 Hz, 2H), 1.78-1.76 (m, 4H), 1.33 (s, 9H); ^{13}CNMR (151

MHz, $CDCl_3$) δ 192.0, 158.3, 155.3, 153.9, 146.9, 142.8, 140.0, 132.0, 131.3, 130.3, 126.8, 124.2, 123.6, 123.3, 120.1, 118.9, 118.8, 110.8, 53.8, 52.6, 43.0, 34.4, 31.5, 29.7, 23.4; IR (neat): 2962, 2875, 2801, 1693, 1608 cm^{-1}.

4. Synthesis of 1 from the β-amino-acid-forming three-component reaction

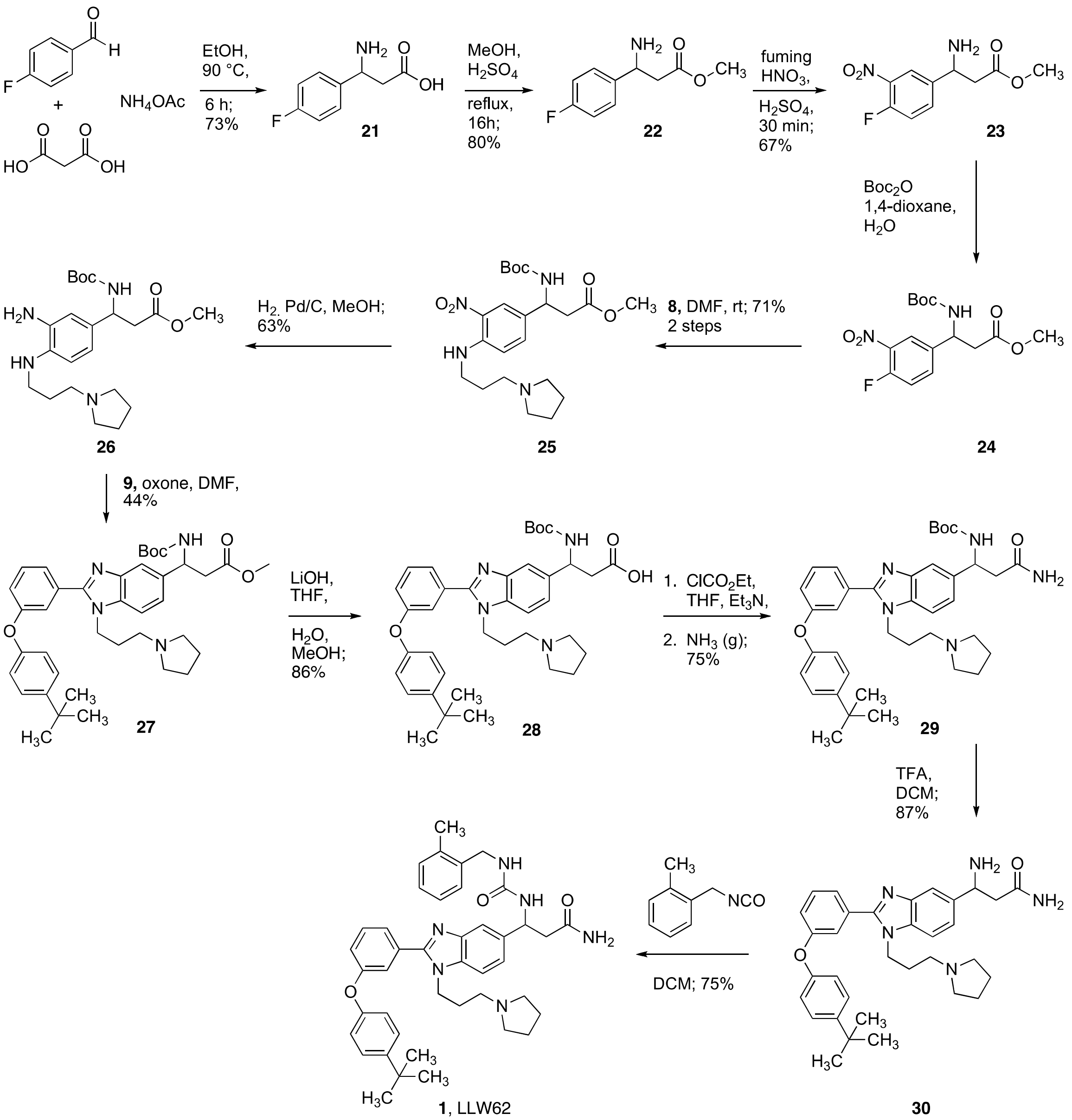

Compound 21

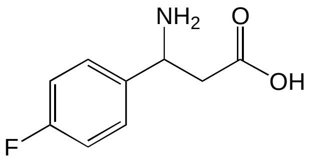

Compound **21** was prepared as described by Tan and Weaver (2002) [2]. To a solution of 4-fluorobenzaldehyde (3.00 g, 24 mmol) in ethanol (5 mL) were added ammonium acetate (4.10 g, 53 mmol) and malonic acid (2.76 g, 27 mmol). The reaction mixture was stirred under ethanol reflux for 6 hours. Then, the system was cooled to room temperature and the precipitate was filtered. The white solid was washed with EtOH (2 × 20 mL) and diethyl ether (3 × 20 mL) yielding 3.25 g (73%) of product. ^{1}H NMR (400 MHz, D_2O/K_2CO_3) δ 7.29 (dd, *J* = 8.8, 5.3 Hz 2H), 7.03 (t, *J* = 8.8 Hz, 2H), 4.46 (t, *J* = 7.3 Hz, 1H), 2.72 (dd, *J* = 16.0, 7.8 Hz, 1H), 2.62 (dd, *J* = 16.0, 6.9 Hz, 1H). ^{1}H NMR spectrum matches those reported in the literature [1].

Compound 22

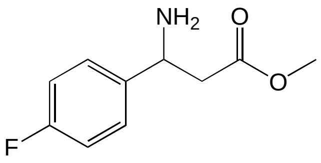

Compound **21** (2.00 g, 11 mmol) was suspended in methanol (40 mL), and concentrated sulfuric acid was added. The resultant solution was stirred at the reflux temperature overnight. Then, after cooling to room temperature, the reaction mixture was neutralized by addition of solid sodium bicarbonate in small portions. MeOH was removed under reduced pressure, and to the residue was added 15 mL of water. The residue was brought to pH 9 by using a 3 M NaOH solution, and then the product was extracted with EtOAc (3 × 20 mL). The organic layers were combined, dried over anhydrous Na_2SO_4, filtered, and then the solvent was removed under reduced pressure. Product was obtained as 1.72 g (80%) of colorless oil. ^{1}H NMR (400 MHz, $CDCl_3$) δ 7.34 (dd, *J* = 5.4, 8.7 Hz, 2H), 7.02 (t, *J* = 8.7 Hz, 2H), 4.43 (t, *J* = 6.8 Hz, 1H), 3.69 (s, 3H), 2.64 (d, *J* = 6.8

Hz, 2H); ^{13}C NMR (101 MHz, $CDCl_3$) δ 172.4, 162.1 (d, *J* = 246.9 Hz), 140.5, 127.9 (d, *J* = 7.7 Hz), 115.5 (d, *J* = 21.4 Hz), 52.1, 51.7, 44.1; IR (neat): 3377, 2956, 1724, 1500 cm^{-1}; HRMS (CI-GC/MS) *m*/*z* calcd for $C_{10}H_{13}FNO_2$ $(M + H)^+$ 198.0930, found 198.0935.

Compound 23

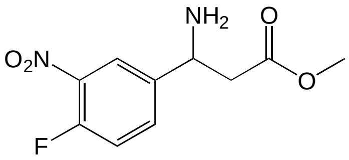

To a solution of concentrated H_2SO_4 (0.75 mL) and fuming HNO_3 (0.1 mL) at 0 °C was added compound **22** (0.300 g, 1.5 mmol) in small portions. The reaction mixture was stirred at 0 °C for 45 min. The reaction mixture was poured into ice, and then neutralized by using NaOH 3 M (up to pH 8). The product was extracted with DCM, then the combined organics were dried over anhydrous Na_2SO_4, filtered and concentrated under reduced pressure yielding 0.250 g (67%) of product as a yellow oil. ^{1}H NMR (400 MHz, $CDCl_3$) δ 8.12 (dd, *J* = 7.1, 2.1 Hz, 1H), 7.69 (m, 1H), 7.28 (m, 1H), 4.52 (t, *J* = 6.7 Hz, 1H), 3.70 (s, 3H), 2.67 (d, *J* = 6.7, 2H), 1.85 (br s, 2H); ^{13}C NMR (101 MHz, $CDCl_3$) δ 171.8, 154.8 (d, *J* = 264.3 Hz), 141.9 (*J* = 4.1 Hz), 137.5, 133.8 (d, *J* = 8.5 Hz), 124.2 (d, *J* = 2.6 Hz), 118.7 (d, *J* = 21.0 Hz), 52.1, 51.6, 43.8; IR (neat): 3386, 2951, 1730, 1533 cm^{-1}; HRMS (ESI) *m*/*z* calcd for $C_{10}H_{12}FN_2O_4$ $(M + H)^+$ 243.0781, found 243.0779.

Compound 24

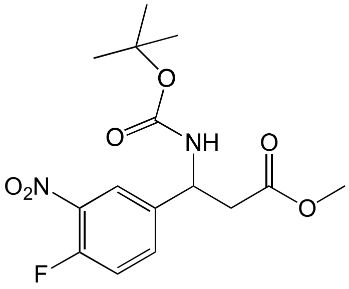

To a solution of the amino compound **23** (1.63 g, 6.7 mmol) in 100 mL of THF/H_2O (1:1 v:v) at 0 °C were added K_2CO_3 (4.63 g, 34 mmol) and Boc_2O (1.46 g, 6.7 mmol). The reaction mixture was allowed to reach room temperature and was stirred overnight. Then, the crude mixture was brought to pH 2.0 by using 1 M HCl solution, and the product was extracted with EtOAc. The combined organic layers were dried over anhydrous Na_2SO_4, filtered and concentrated under reduced pressure. **24** was obtained as a pale yellow oil (1.40 g, 61%). ^{1}H NMR (600 MHz, $CDCl_3$, 50 °C) δ 8.00 (dd, *J* = 6.9, 2.5 Hz, 1H), 7.59 (m, 1H), 7.25 (m, 1H), 5.59 (br s, 1H), 5.10 (m, 1H), 3.65 (s, 3H), 2.85 (m, 2H), 1.42 (s, 9H); ^{13}C NMR (151 MHz, $CDCl_3$, 50 °C) δ 170.6, 154.8, 154.6 (d, *J* = 264.8 Hz) 138.9, 137.4, 133.2 (d, *J* = 8.5 Hz), 123.7 (d, *J*=2.6 Hz), 118.5 (d, *J* = 21.1 Hz), 80.4, 51.9, 50.3, 40.0, 28.2; IR (neat): 3429, 2890, 1710, 1548 cm^{-1}; HRMS (ESI) *m*/*z* calcd for $NaC_{15}H_{19}FN_2O_6$ $(M + Na)^+$ 365.1125, found 365.1127.

Compound 25

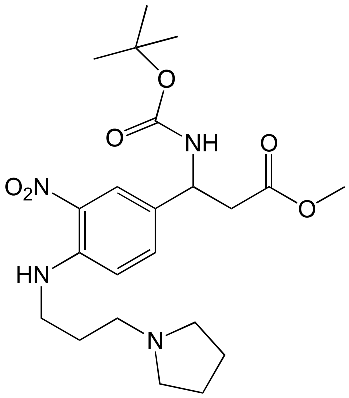

To a solution of compound **24** (0.520 g, 1.5 mmol) in 3.5 mL DCM was added K_2CO_3 (0.622 g, 4.5 mmol) and *N*-(3-aminopropyl)pyrrolidine (0.228 mL, 1.8 mmol) at room temperature, and the reaction was stirred overnight. Water (10 mL) was added to the reaction and the mixture was extracted with DCM (3 × 10 mL). The combined extracts were dried over anhydrous Na_2SO_4, filtered and concentrated. Silica gel chromatography (95:5 DCM:MeOH + 1% TEA) resulted in an orange oil (75%, .509 g). ^{1}H NMR (600 MHz, $CDCl_3$, 50 °C) δ 8.30 (s, 1H), 8.08 (d, *J* = 2.3 Hz, 1H), 7.38 (dd, *J* = 8.8, 2.3 Hz, 1H), 6.85 (d, *J* = 8.9 Hz, 1H),

5.35 (s, 1H), 4.98 (m, 1H), 3.64 (s, 3H), 3.39 (m, 2H), 2.81 (m, 2H), 2.62 (t, *J* = 6.7 Hz, 2H), 2.56 (br s, 4H), 1.91 (q, *J* = 6.7 Hz, 2H), 1.82 (m, 4H), 1.43 (s, 9H); ^{13}C NMR (151 MHz, $CDCl_3$, 50 °C) δ 171.0, 154.9, 144.9, 134.5, 131.7, 128.3, 123.9, 114.2, 79.9, 54.2, 54.1, 51.7, 50.3, 42.0, 40.3, 28.3, 27.8, 23.5; IR (neat): 3440, 3388, 2973, 2802, 1707, 1636 cm^{-1}; HRMS (ESI) *m*/*z* calcd for $NaC_{22}H_{34}N_4O_6$ $(M + Na)^+$ 473.2376, found 473.2368.

Compound 26

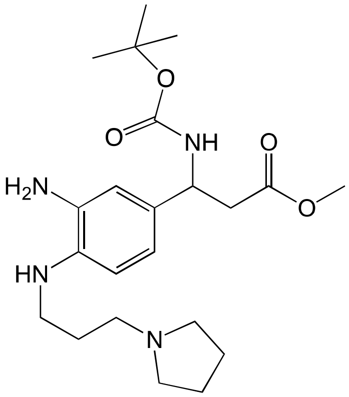

To an argon-flushed flask containing compound **25** (0.585 g, 1.3 mmol) in MeOH (7.0 mL) was added Pd/C (10%, 0.891 g, 0.78 mmol) and the solution was stirred. The atmosphere was exchanged with H_2(g) and stirred overnight. The reaction mixture was filtered through a pad of celite and concentrated to yield the crude product. The crude mixture was purified by silica gel chromatography (90:10 DCM:MeOH, 1% TEA) to yield **26** (0.345 g, 63%) as a purple oil. ^{1}H NMR (600 MHz, $CDCl_3$, 50 °C) δ 6.67 (dd, *J* = 8.1, 2.1 Hz, 1H), 6.60 (d, *J* = 2.1 Hz, 1H), 6.53 (d, *J* = 8.1 Hz, 1H), 5.15 (br s, 1H), 4.93 (m, 1H), 3.60 (s, 3H), 3.19 (t, *J* = 6.4 Hz, 2H), 2.76 (m, 2H), 2.70 (t, *J* = 6.7 Hz, 2H), 2.64 (s, 4H), 1.90 (p, *J* = 6.6 Hz, 2H), 1.83 (m, 4H), 1.42 (s, 9H); ^{13}C NMR (151 MHz, $CDCl_3$, 50 °C) δ 171.39, 154.97, 137.10, 134.45, 130.99, 117.69, 114.09, 111.26, 79.28, 54.87, 54.11, 51.42, 51.41, 43.39, 41.02, 28.34, 27.64, 23.46; IR (neat): 3450, 2977, 2809, 2368, 1710 cm^{-1}; HRMS (ESI) *m*/*z* calcd for $NaC_{22}H_{36}N_4O_4$ $(M + Na)^+$ 443.2634, found 443.2625.

Compound 27

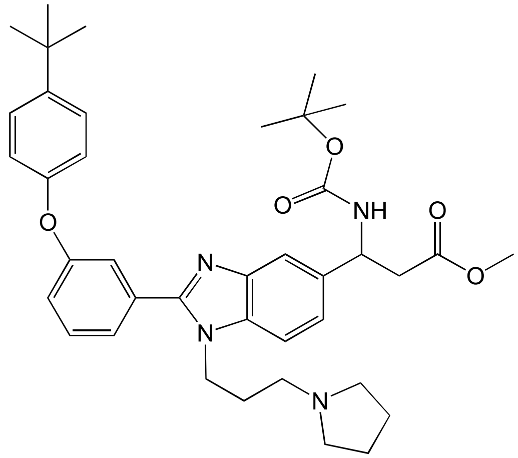

To a solution of compound **26** (0.674g, 1.60 mmol) and 3-(4-*tert*-butylphenoxy)benzaldehyde (0.407g, 1.60 mmol) in DMF (10 mL) and H_2O (0.4 mL) was added Oxone (0.590g, 0.96mmol) in small portions. The reaction was stirred overnight open to air at room temperature. Water was added, and the reaction was extracted with DCM. The organic layer was washed several times with H_2O. Purification was performed by silica gel chromatography with 90:10 DCM:MeOH + 1% TEA as the eluent. The product was obtained as a pale yellow oil (0.463 g, 44%). ^{1}H NMR (600 MHz, $CDCl_3$, 50 °C) δ 7.70 (s, 1H), 7.43 (m, 2H), 7.36 (m, 4H) 7.25 (m, 1H), 7.13 (m, 1H), 6.98 (d, *J* = 8.4 Hz, 2H), 5.37 (br s, 1H), 5.23 (m, 1H), 4.29 (m, 2H), 3.61 (s, 3H), 2.91 (m, 2H), 2.38 (s, 2H), 2.36 (t, *J* = 6.7 Hz, 4H), 1.91 (p, *J* = 6.9 Hz, 2H), 1.72 (s, 4H), 1.42 (s, 9H), 1.32 (s, 9H); ^{13}C NMR (151 MHz, $CDCl_3$, 50 °C) δ 171.2, 158.0, 155.0, 154.2, 153.7, 146.7, 143.3, 135.9, 135.2, 132.3, 129.9, 126.6, 123.7, 121.6, 119.7, 119.1, 118.8, 117.1, 110.2, 79.5, 53.9, 52.9, 51.5, 51.5, 42.9, 41.2, 34.3, 31.4, 29.0, 28.3, 23.5; IR (neat): 3436, 3053, 2967, 2797, 2348, 1707 cm^{-1}; HRMS (ESI) *m*/*z* calcd for $NaC_{39}H_{50}N_4O_5$ $(M + Na)^+$ 667.3679, found 677.3694.

Compound 28

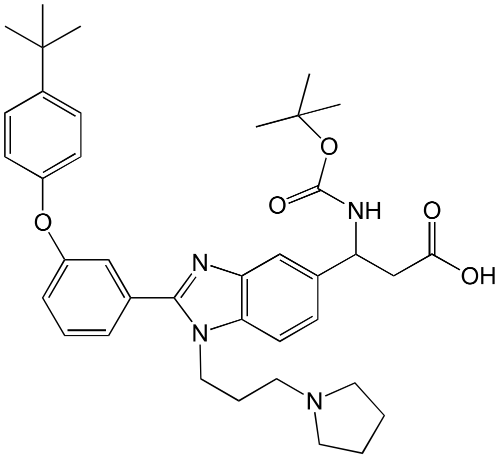

The imidazole compound **27** (0.250 g, 0.35 mmol) was dissolved in MeOH, THF and H_2O (5.3 mL of a 1:1.5:1 mixture) and cooled to 0 °C. LiOH (0.033 g, 1.4 mmol) was added and reaction was allowed to come to room temperature overnight. The solvent was evaporated, then the crude product was rediluted in H_2O (5 mL). The pH was brought to ~7 and a white precipitate was observed. The product was extracted with EtOAc, then the combined organics were dried, filtered and concentrated to yield **28** (0.195 g, 86%). The product was used without further purification. ^{1}H NMR (600 MHz, $CDCl_3$, 50 °C) δ 7.79 (s, 1H), 7.31 (d, *J* = 8.7 Hz, 2H), 7.22 (m, 3H), 7.10 (br s, 1H), 6.95 (d, *J* = 8.9 Hz, 1H), 6.89 (d, *J* = 8.7 Hz, 2H), 6.80 (br s, 1H), 6.64 (br s, 1H), 5.18 (br s, 1H), 3.76 (br s, 2H), 2.90 (m, 2H), 2.75 (br s, 4H), 2.55 (m, 2H), 1.85 (s, 4H), 1.56 (br s, 2H), 1.43 (s, 9H), 1.31 (s, 9H); ^{13}C NMR (151 MHz, $CDCl_3$, 50 °C) δ 176.68, 157.77, 155.43, 154.25, 152.25, 146.50, 142.53, 137.15, 134.37, 131.99, 129.63, 126.53, 123.41, 122.07, 119.27, 119.18, 118.69, 116.67, 110.26, 78.87, 52.88, 51.97, 42.45, 41.68, 34.26, 31.42, 28.48, 26.83, 23.08; IR (neat): 3382, 2968, 1704, 1587 cm^{-1}; HRMS (ESI) *m*/*z* calcd for $NaC_{38}H_{48}N_4O_5$ $(M + Na)^+$ 663.3522, found 663.3519.

Compound 29

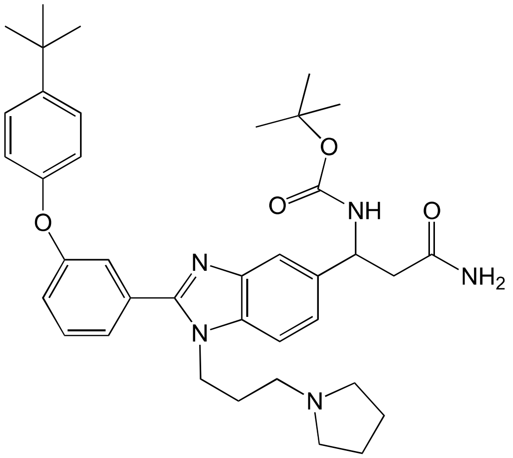

The carboxylic acid compound **28** (0.366 g, 0.57 mmol) was dissolved in THF (5.7 mL) and cooled to −42 °C. TEA (0.116 g, 1.1 mmol) was added, followed by ethyl chloroformate (0.093 g, 0.86 mmol). The reaction was stirred for 1 hour, and then NH_3(g) was bubbled into the solution for 30 minutes while coming to room temperature. The reaction mixture was stirred overnight at room temperature. The crude mixture was quenched with ethanol (5 mL) and the solvent was evaporated. The residue was taken up in H_2O and DCM. The product was extracted with DCM, and the combined organic layers were dried, filtered and concentrated to yield 0.239 g (65%) of product as a white solid. ^1H NMR (600 MHz, $CDCl_3$, 50 °C) δ 7.71 (s, 1H), 7.45-7.36 (m, 3H), 7.35 (m, 2H), 7.32 (m, 1H), 7.27 (m, 1H), 7.12 (m, 1H), 6.97 (m, 2H), 5.91 (br s, 1H), 5.85 (d, *J* = 8.1 Hz, 1H), 5.35 (br s, 2H), 5.17 (m, 1H), 4.29 (m, 2H), 2.79 (m, 2H), 2.40 (br s, 4H), 2.37 (t, *J* = 6.8 Hz, 2H), 1.92 (p, *J* = 6.9 Hz, 2H), 1.73 (m, 4H), 1.40 (s, 9H), 1.32 (s, 9H); ^{13}C NMR (151 MHz, $CDCl_3$, 50 °C) δ 172.55, 158.05, 155.41, 154.20, 153.62, 146.74, 143.25, 135.16, 132.19, 129.94, 126.63, 123.70, 121.54, 119.66, 119.08, 118.76, 117.07, 110.24, 79.57, 53.85, 52.85, 46.11, 42.89, 42.71, 34.28, 31.40, 28.90, 28.34, 23.47; IR (neat): 3053, 2970, 1696 cm^{-1}; mp 141.0–142.5 °C; HRMS (ESI) *m/z* calcd for $C_{38}H_{50}N_5O_4$ $(M + H)^+$ 640.3863, found 640.3846.

Compound 30

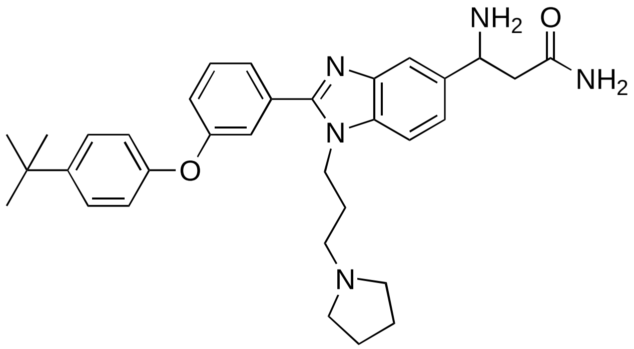

To a solution of the compound **29** (0.118 g, 0.18 mmol) in anhydrous DCM (3 mL) at 0 °C was added trifluoroacetic acid (0.6 mL). The reaction mixture was allowed to reach room temperature and was stirred for 3 h. Then, a saturated aqueous solution of sodium bicarbonate (5 mL) was added and the crude product was extracted with DCM. The solvent was removed under reduced pressure yielding 0.087 g (87%) of product as a slightly yellow solid. The compound was submitted to the next step without further purification.

Compound 1 (LLW62)

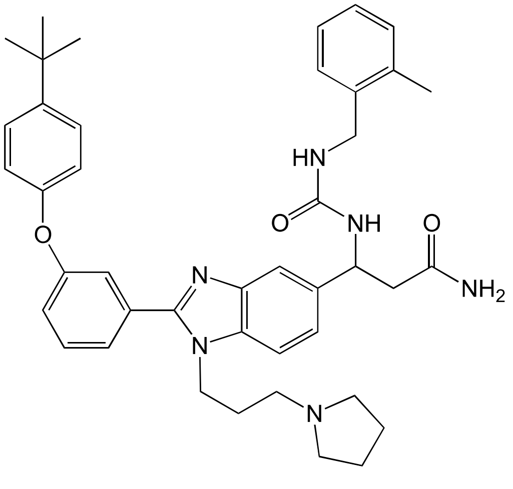

To a solution of compound **30** (0.087 g, 0.16 mmol) in a mixture of acetonitrile (2 mL) and DCM (1 mL), was added 2-methylbenzyl isocyanate (0.0248 g, 0.193 mmol). The reaction mixture was stirred at room temperature for 2 hours. Then,

the solvent was removed under reduced pressure, and the product was purified by flash chromatography using a mixture of 95:5 DCM:MeOH w/1% TEA as the eluent. The product was obtained as a white solid (0.083 g, 75%). ^{1}H NMR (600 MHz, $CDCl_3$, 50 °C) δ 7.72 (br s, 1H), 7.39 (t, J = 7.9 Hz, 1H), 7.34 (m, 4H), 7.26 (m, 2H), 7.09 (m, 2H), 7.03 (m, 3H), 6.95 (m, 2H), 6.58 (s, 1H), 6.38 (d, J = 8.1 Hz, 1H), 5.47 (s, 1H), 5.33 (m, 1H), 4.23 (m, 4H), 2.75 (m, 2H), 2.37 (m, 7H), 2.18 (s, 3H), 1.88 (m, 2H), 1.71 (m, 4H), 1.31 (s, 9H); ^{13}C NMR (151 MHz, $CDCl_3$, 50 °C) δ 173.6, 158.3, 158.2, 154.4, 153.8, 147.1, 143.3, 137.3, 137.2, 136.2, 135.2, 132.2, 130.4, 130.2, 127.9, 127.3, 126.9, 126.2, 123.9, 122.2, 119.9, 119.3, 119.0, 117.0, 110.6, 54.1, 53.0, 52.0, 43.6, 43.1, 42.5, 34.5, 31.7, 29.0, 23.7, 19.0; IR (neat): 3315, 2965, 1681 cm^{-1}; mp 111.1–112.0 °C; HRMS (ESI) *m/z* calcd for $NaC_{42}H_{50}N_6O_3$ $(M + Na)^+$ 709.3842, found 709.3843.

5. References

1. Dietrich, S. A.; Lindauer, R.; Stierlin, C.; Gertsch, J.; Matesanz, R.; Notararigo, S.; Diaz, J. F.; Altmann, K.-H. *Chemistry--A European Journal* **2009**, *15*, 10144.

2. Tan, C. Y. K.; Weaver, D. F. *Tetrahedron* **2002**, *58*, 7449.

6. ^{1}H and ^{13}C NMR spectra

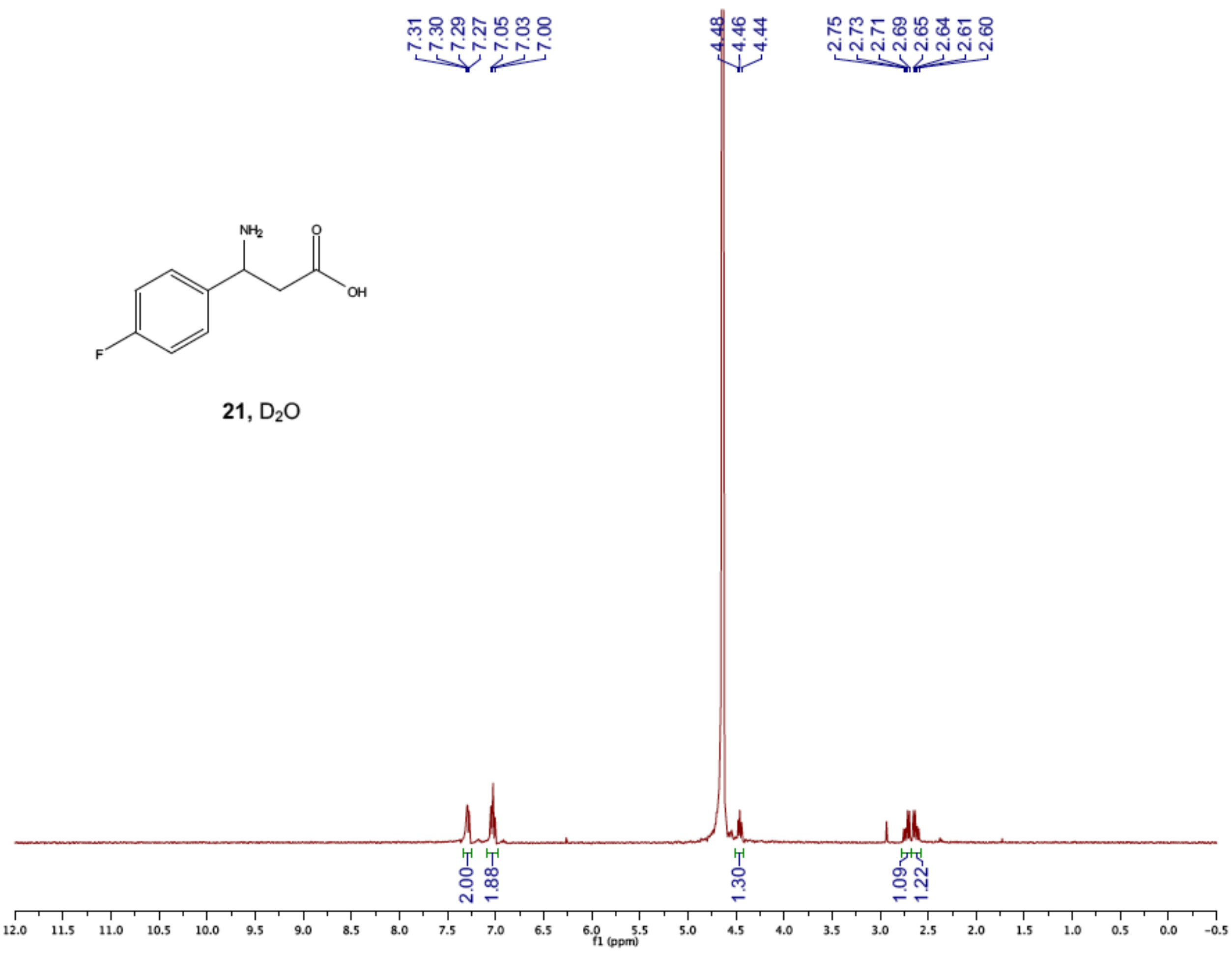
7.31
7.30
7.29
7.27
7.05
7.03
7.00
4.48
4.46
4.44
2.75
2.73
2.71
2.69
2.65
2.64
2.61
2.60
NH2
O
OH
F
21, D2O
2.00
1.88
1.30
1.09
1.22
12.0
11.5
11.0
10.5
10.0
9.5
9.0
8.5
8.0
7.5
7.0
6.5
6.0
5.5
5.0
4.5
4.0
3.5
3.0
2.5
2.0
1.5
1.0
0.5
0.0
-0.5
f1 (ppm)

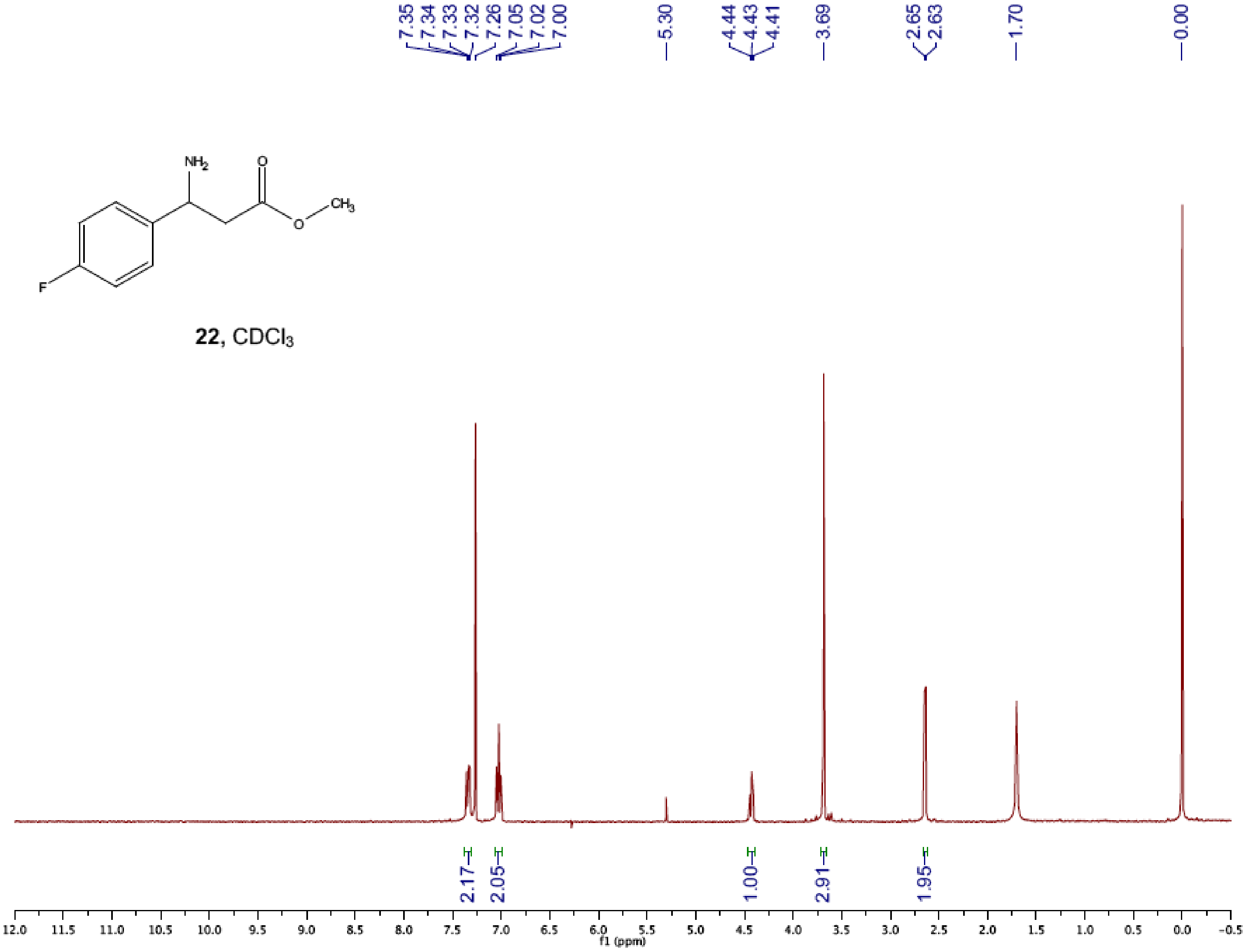
7.35
7.34
7.33
7.32
7.26
7.05
7.02
7.00
5.30
4.44
4.43
4.41
3.69
2.65
2.63
1.70
0.00
NH2
O
O
CH3
F
2.17
2.05
1.00
2.91
1.95
f1 (ppm)

22, $CDCl_3$

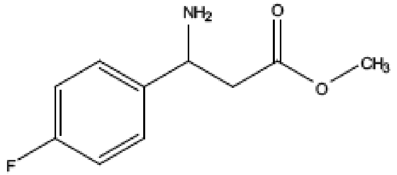
NH2
O
CH3
O
F

22, $CDCl_3$

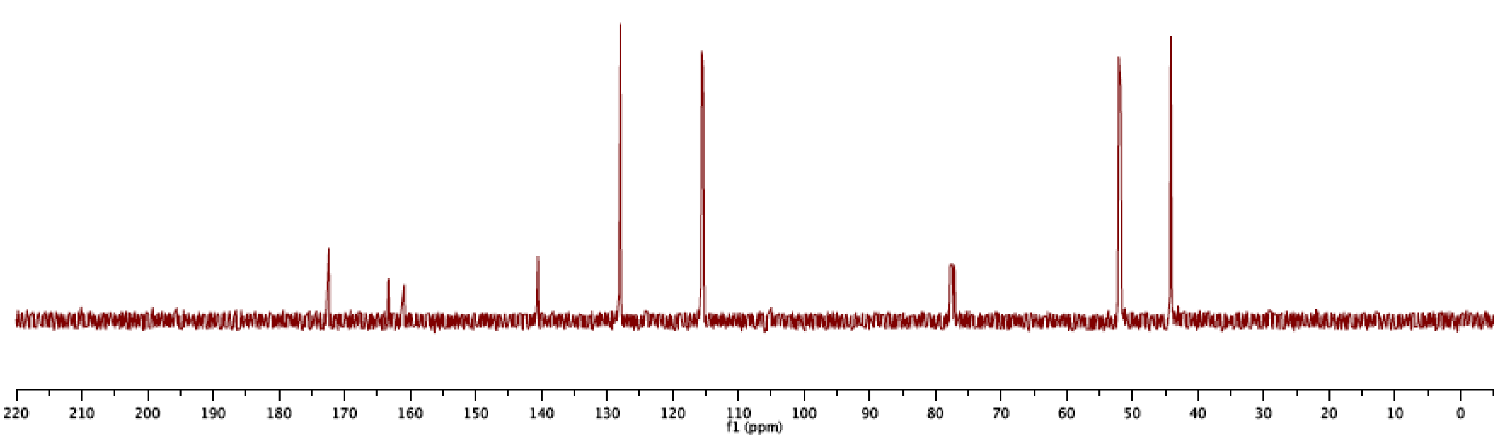
172.4
170.0
163.3
160.9
140.5
128.0
127.9
115.6
115.4
77.7
77.4
77.1
52.1
51.8
44.1
220
210
200
190
180
170
160
150
140
130
120
110
100
90
80
70
60
50
40
30
20
10
0
f1 (ppm)

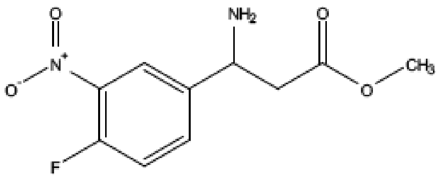

23, $CDCl_3$

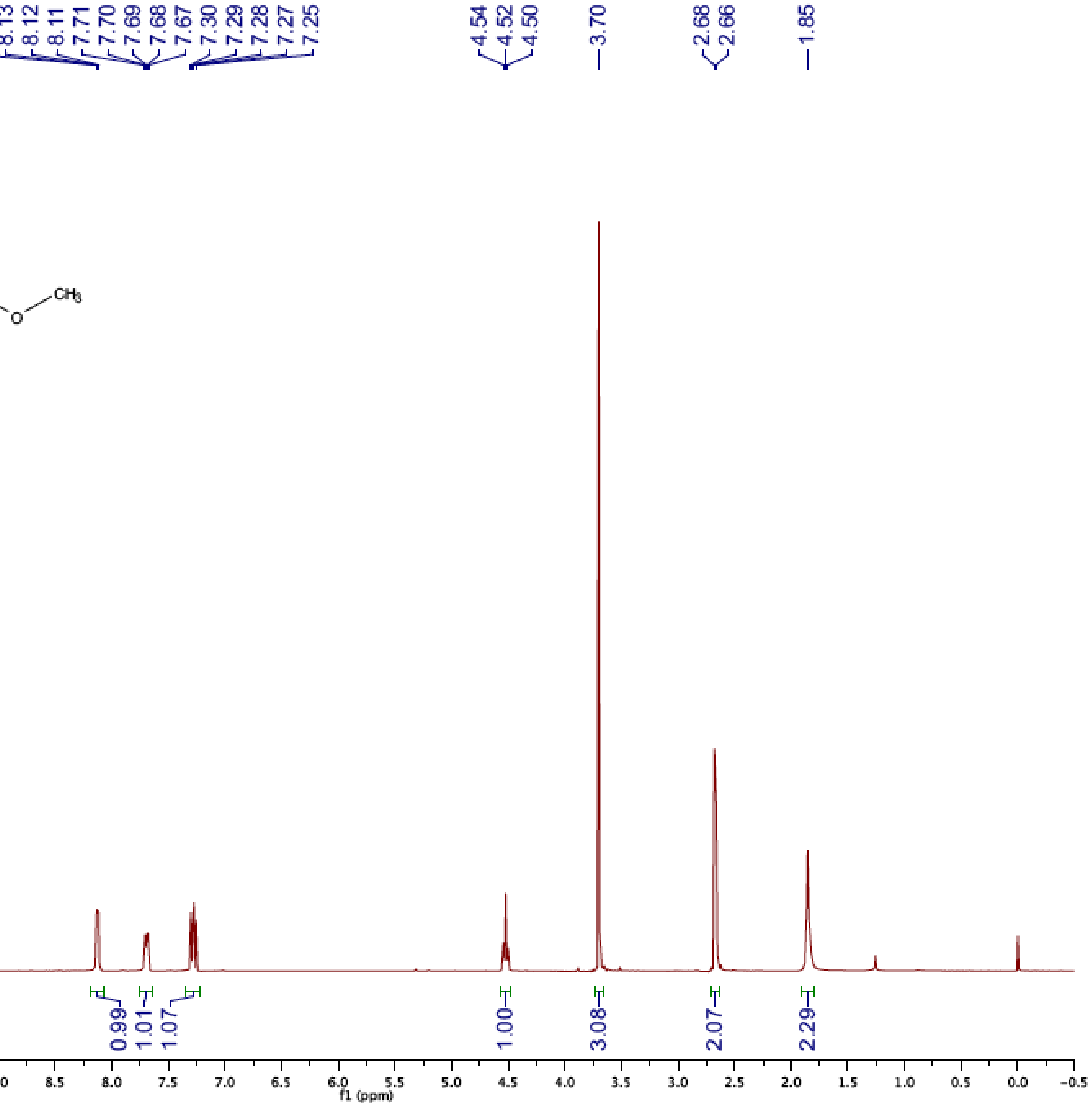
8.13
8.13
8.12
8.11
7.71
7.70
7.69
7.68
7.67
7.30
7.29
7.28
7.27
7.25
4.54
4.52
4.50
3.70
2.68
2.66
1.85
0.99
1.01
1.07
1.00
3.08
2.07
2.29
12.0
11.5
11.0
10.5
10.0
9.5
9.0
8.5
8.0
7.5
7.0
6.5
6.0
5.5
5.0
4.5
4.0
3.5
3.0
2.5
2.0
1.5
1.0
0.5
0.0
-0.5
f1 (ppm)

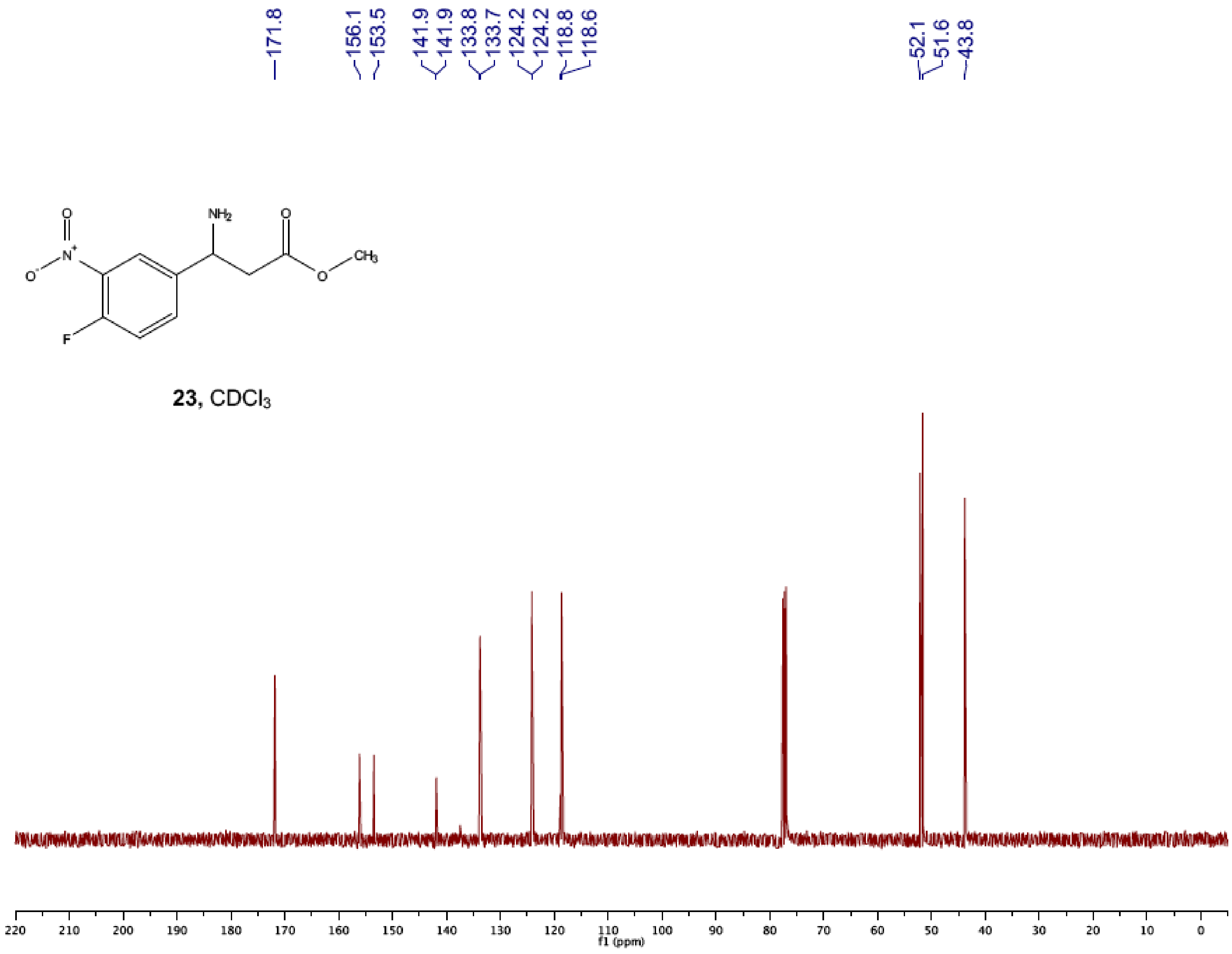
171.8
156.1
153.5
141.9
141.9
133.8
133.7
124.2
124.2
118.8
118.6
52.1
51.6
43.8
f1 (ppm)

23, $CDCl_3$

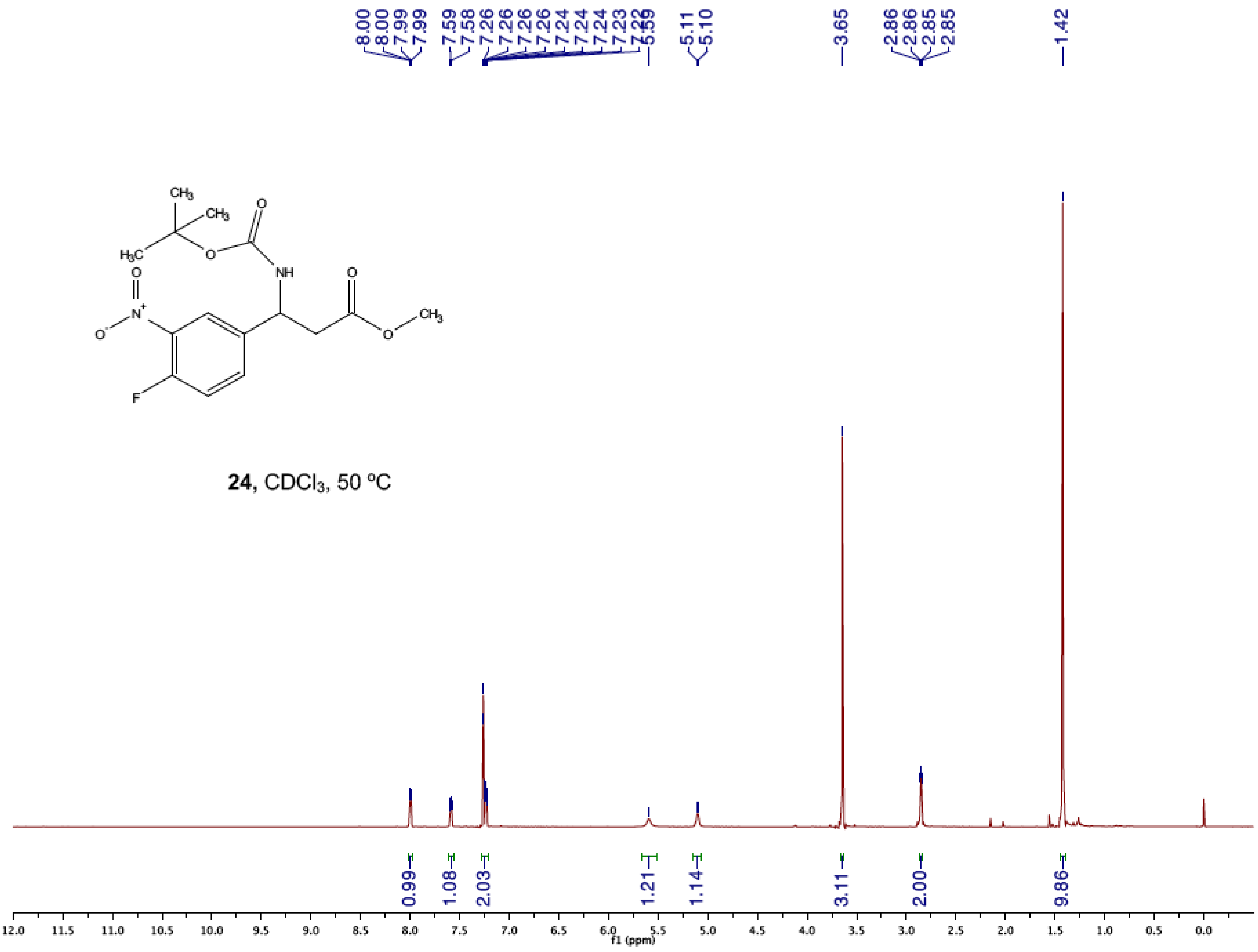

24, $CDCl_3$, 50 ºC

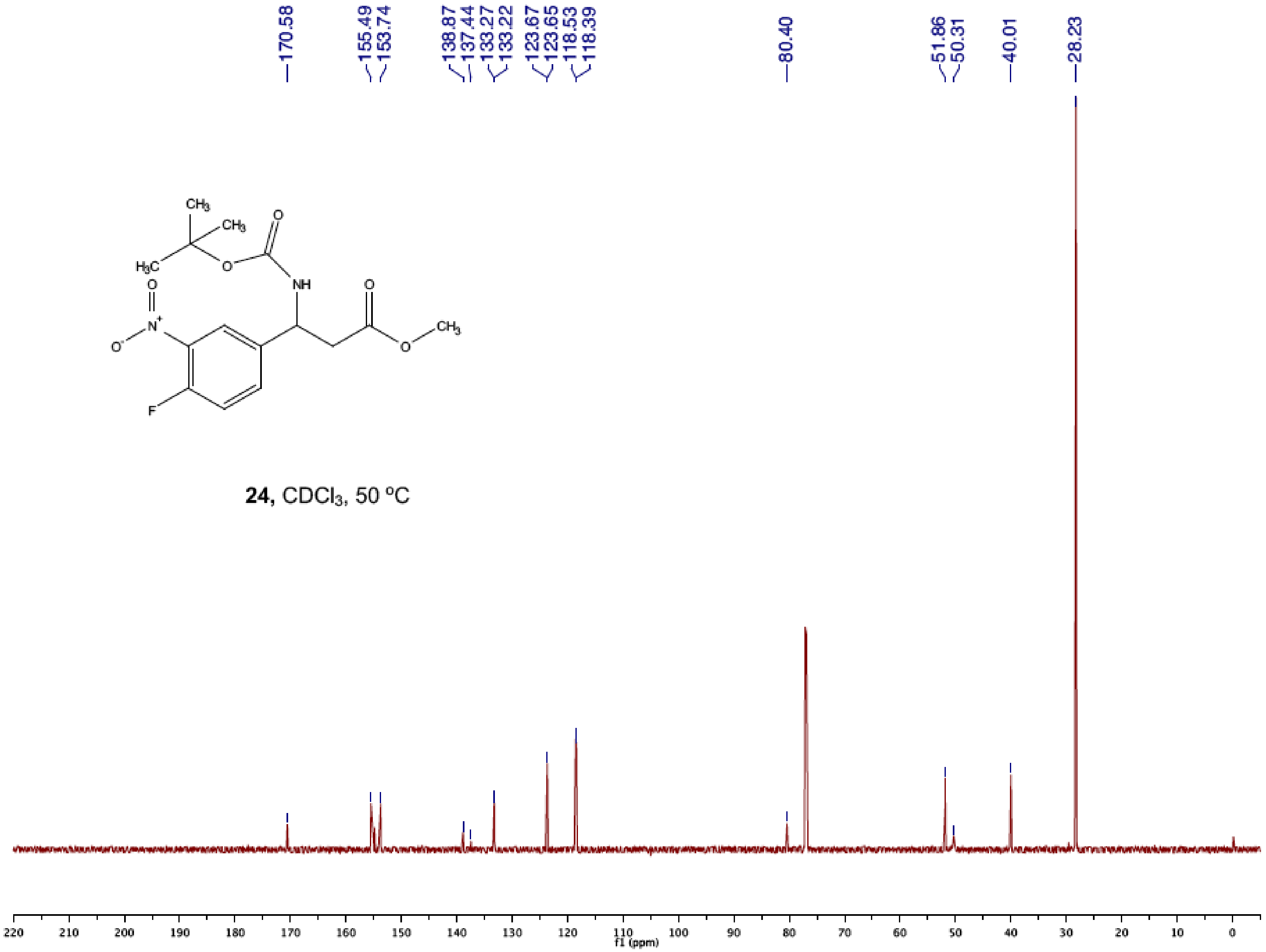

24, $CDCl_3$, 50 ºC

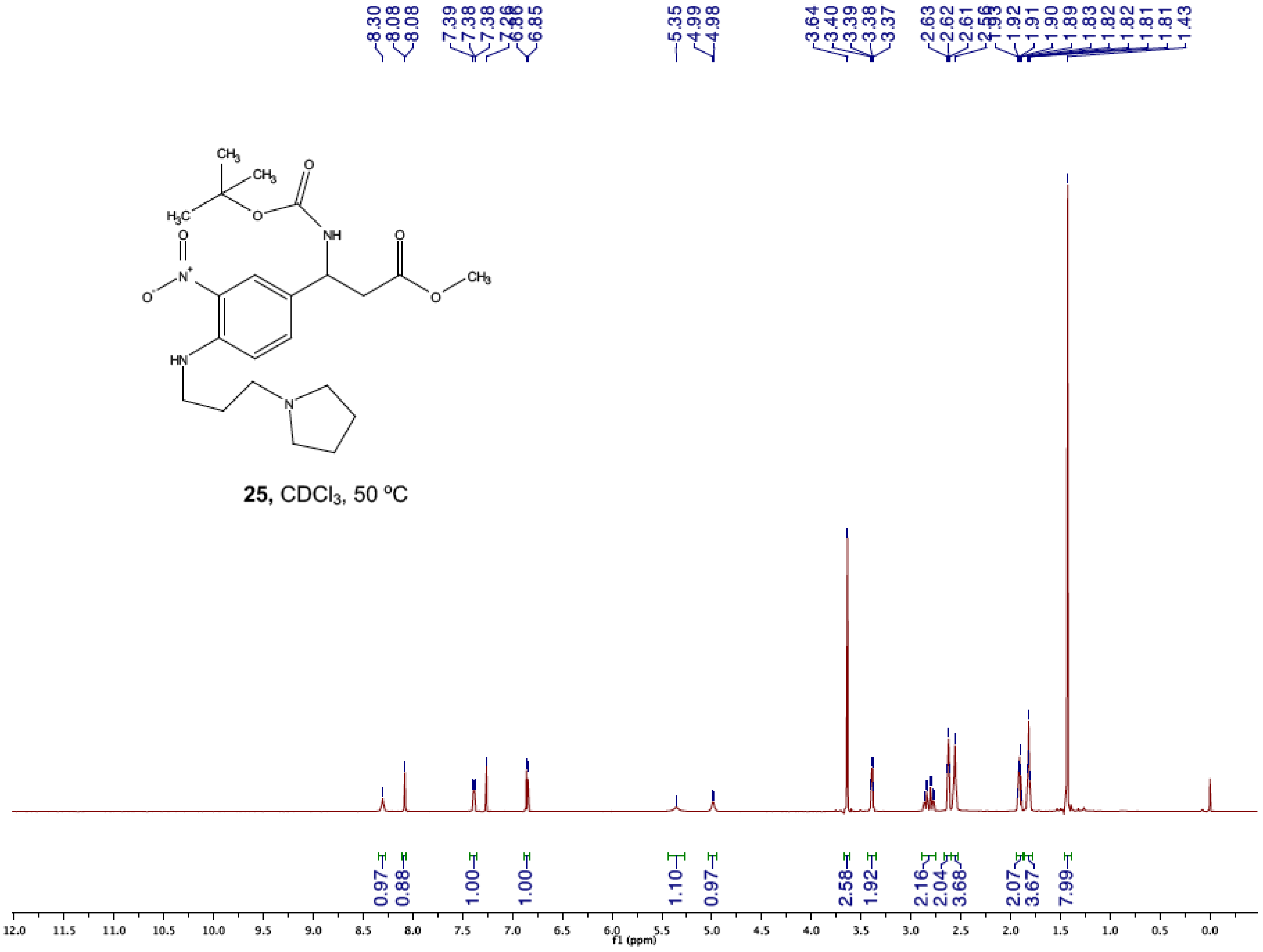

25, $CDCl_3$, 50 °C

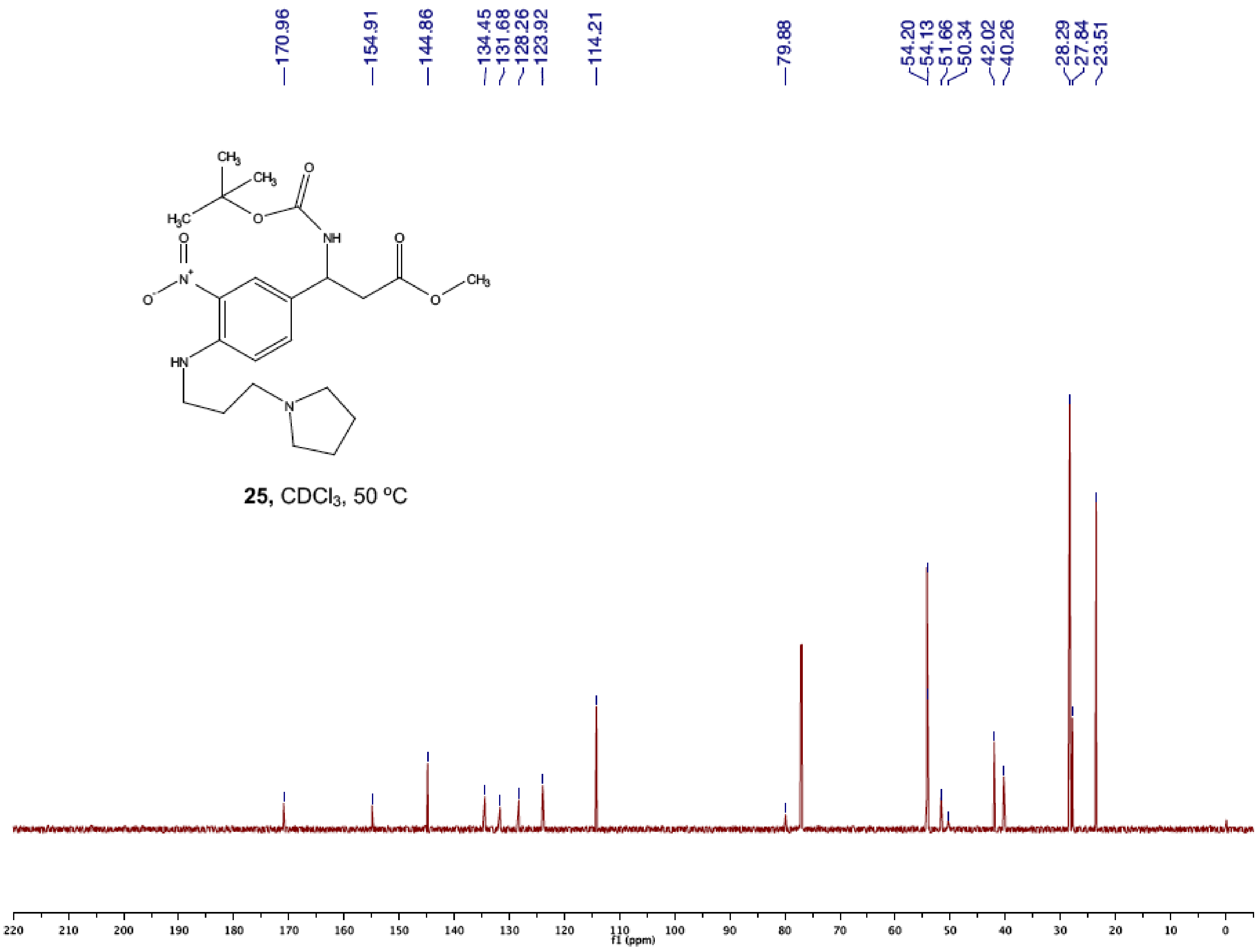
170.96
154.91
144.86
134.45
131.68
128.26
123.92
114.21
79.88
54.20
54.13
51.66
50.34
42.02
40.26
28.29
27.84
23.51
f1 (ppm)

25, $CDCl_3$, 50 °C

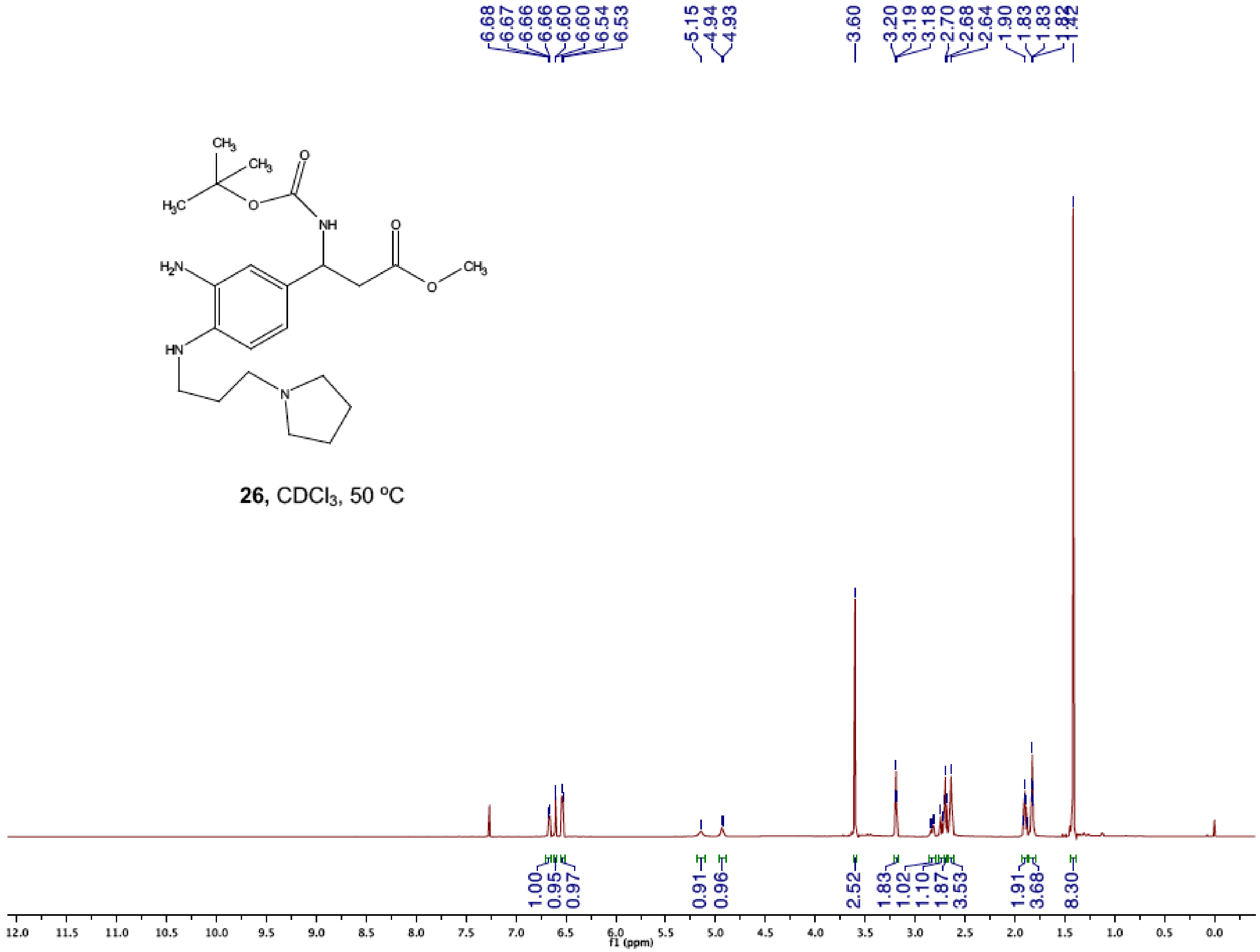

26, $CDCl_3$, 50 ºC

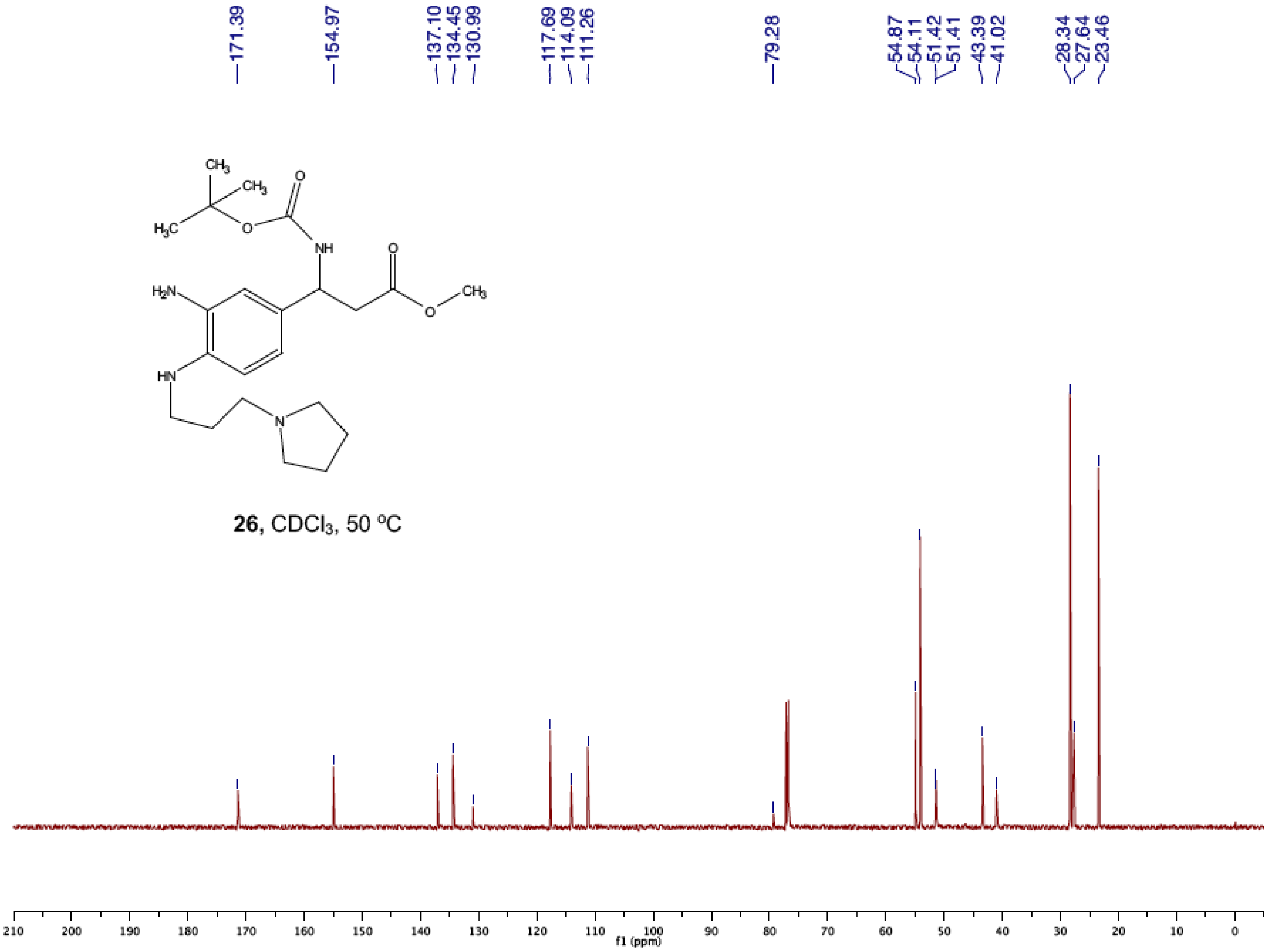
171.39
154.97
137.10
134.45
130.99
117.69
114.09
111.26
79.28
54.87
54.11
51.42
51.41
43.39
41.02
28.34
27.64
23.46
f1 (ppm)

26, $CDCl_3$, 50 °C

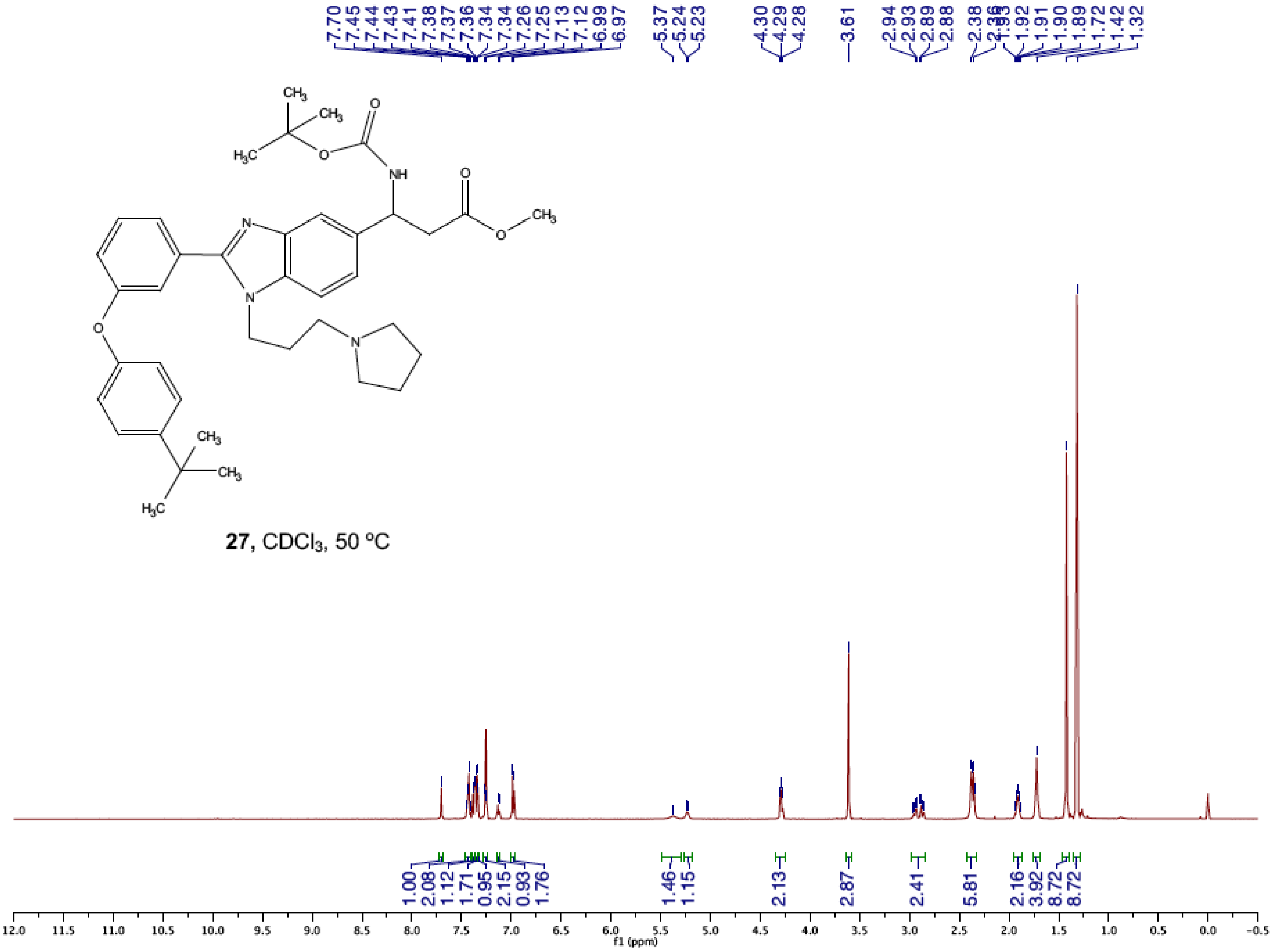
7.70 7.45 7.44 7.43 7.41 7.38 7.37 7.36 7.34 7.34 7.26 7.25 7.13 7.12 6.99 6.97
5.37 5.24 5.23
4.30 4.29 4.28
3.61
2.94 2.93 2.89 2.88
2.38 1.92 1.91 1.90 1.89 1.72 1.42 1.32
1.00 2.08 1.12 1.71 0.95 2.15 0.93 1.76
1.46 1.15
2.13
2.87
2.41
5.81
2.16 3.92 8.72 8.72
f1 (ppm)

27, $CDCl_3$, 50 °C

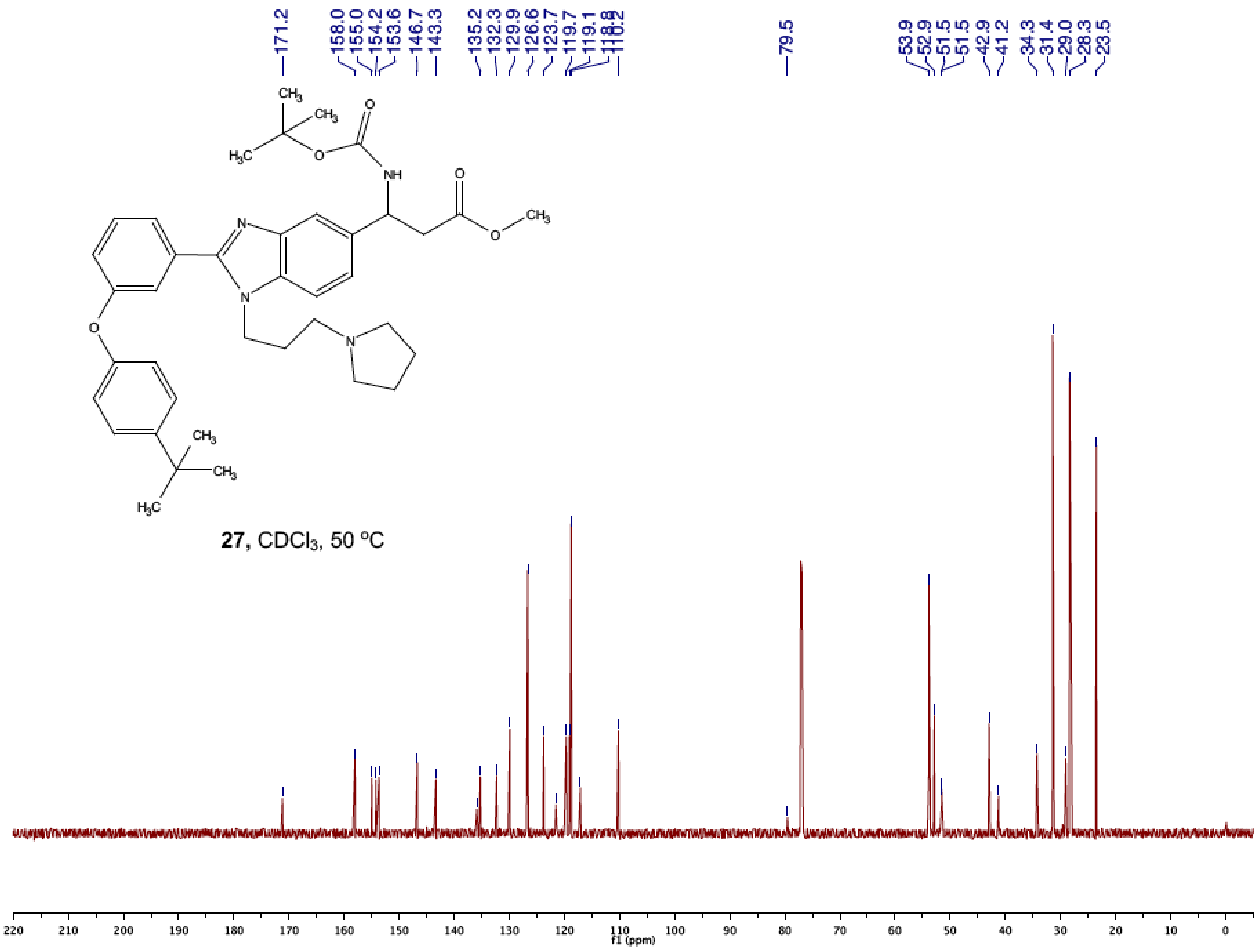
171.2
158.0
155.0
154.2
153.6
146.7
143.3
135.2
132.3
129.9
126.6
123.7
119.7
119.1
118.8
110.2
79.5
53.9
52.9
51.5
51.5
42.9
41.2
34.3
31.4
29.0
28.3
23.5
f1 (ppm)

27, $CDCl_3$, 50 °C

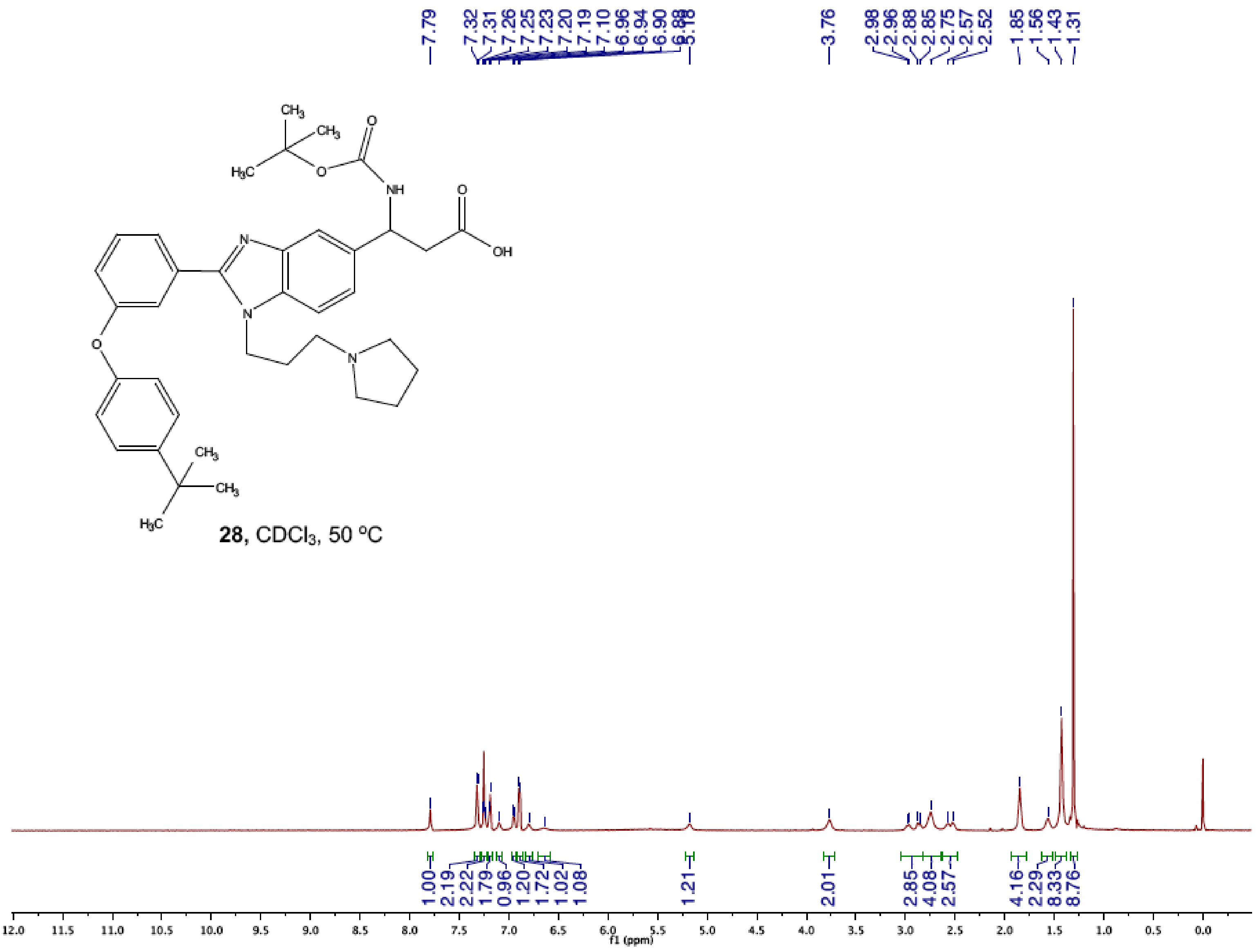
28, CDCl3, 50 °C
f1 (ppm)

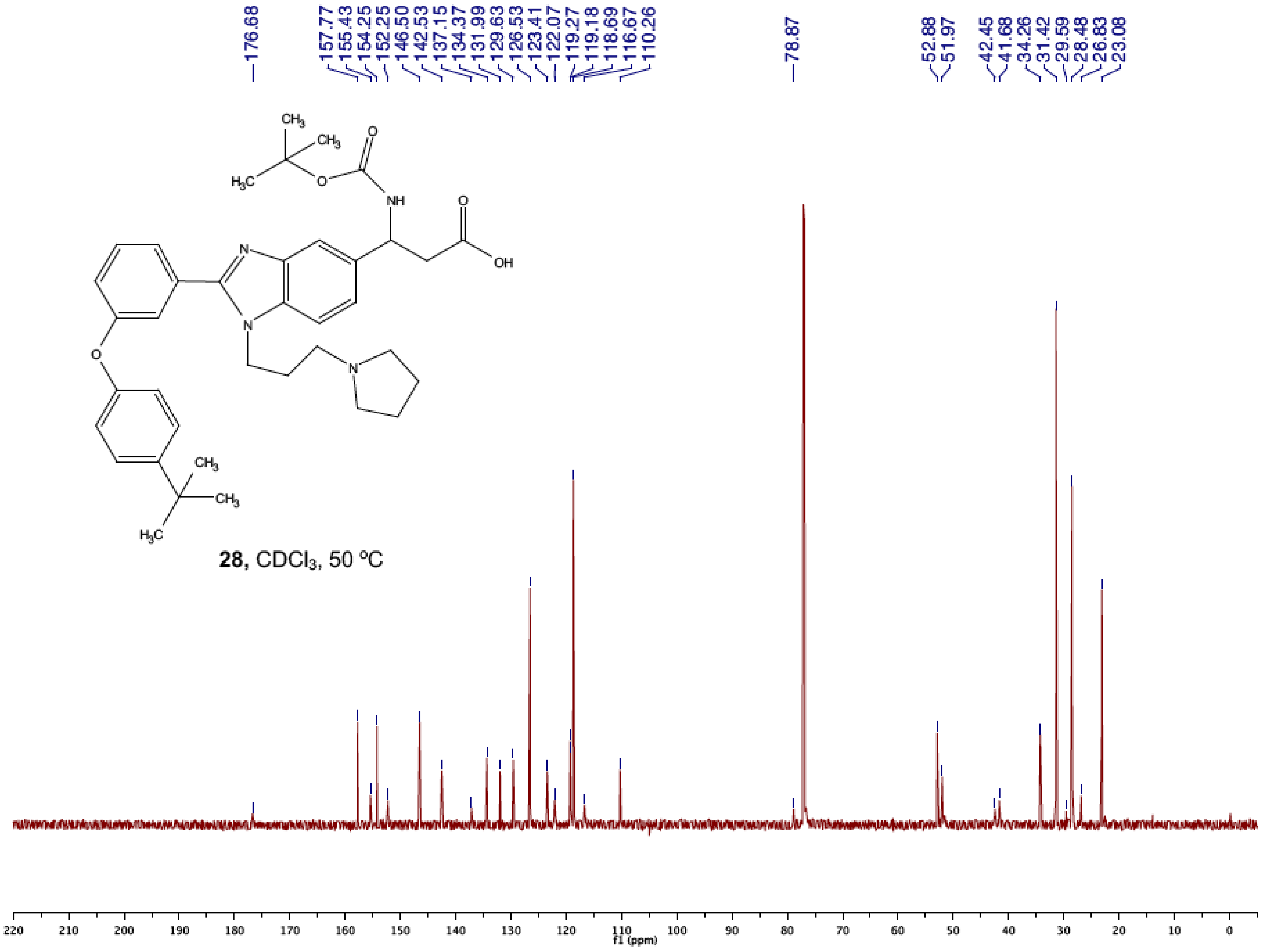
176.68
157.77
155.43
154.25
152.25
146.50
142.53
137.15
134.37
131.99
129.63
126.53
123.41
122.07
119.27
119.18
118.69
116.67
110.26
78.87
52.88
51.97
42.45
41.68
34.26
31.42
29.59
28.48
26.83
23.08
CH3
H3C
O
NH
OH
N
28, CDCl3, 50 ºC
f1 (ppm)

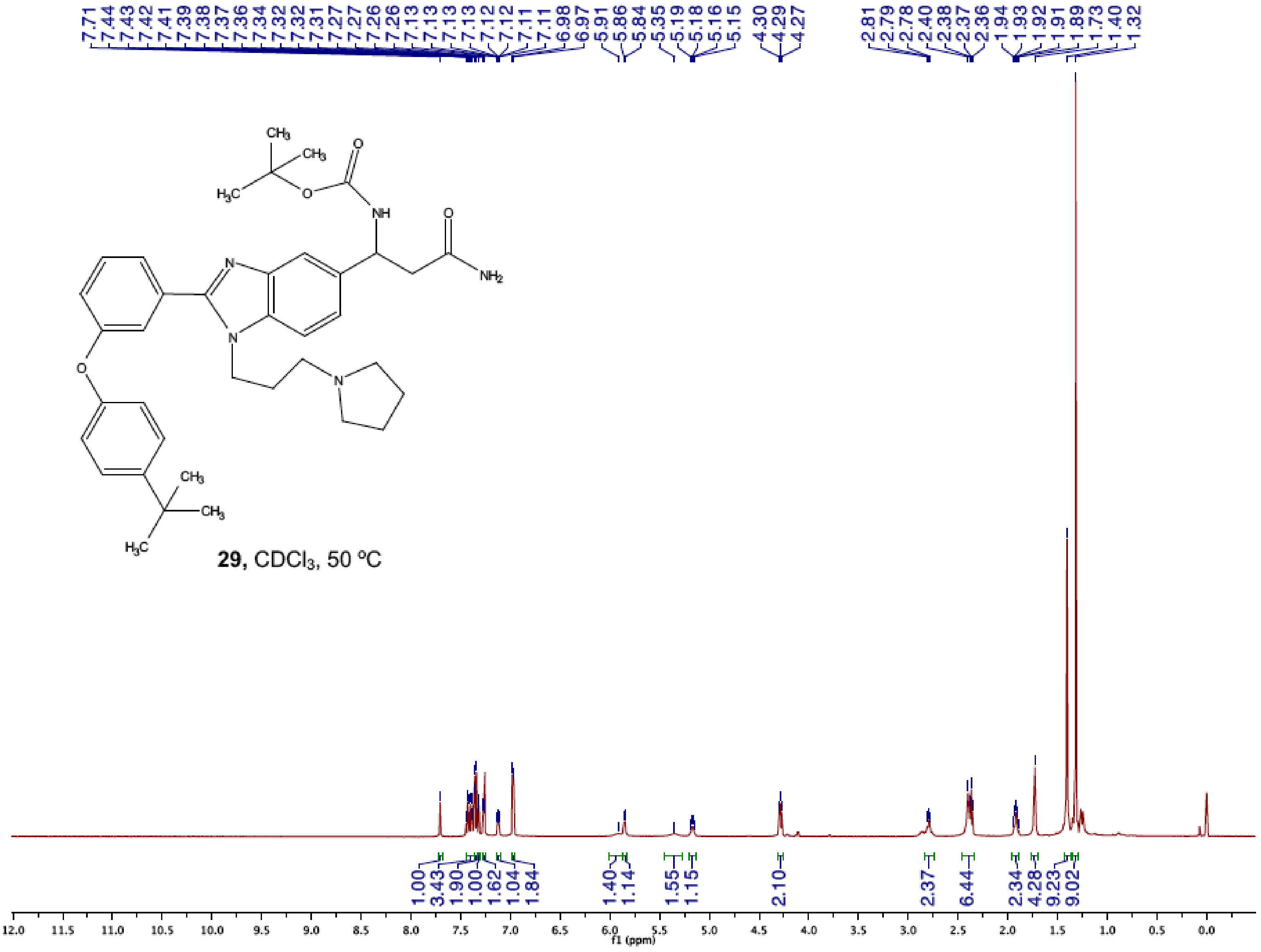

29, $CDCl_3$, 50 ºC

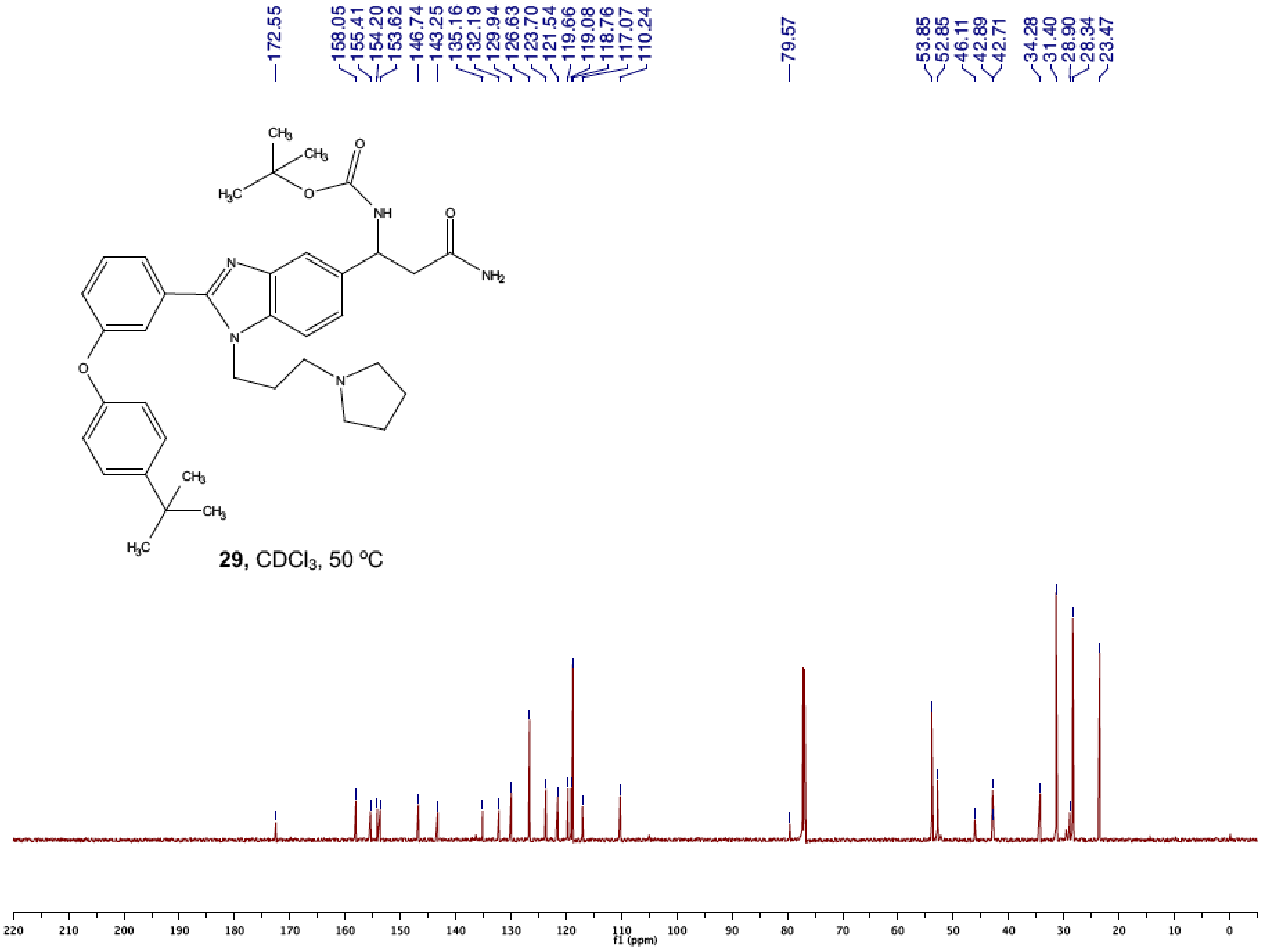

29, $CDCl_3$, 50 ºC

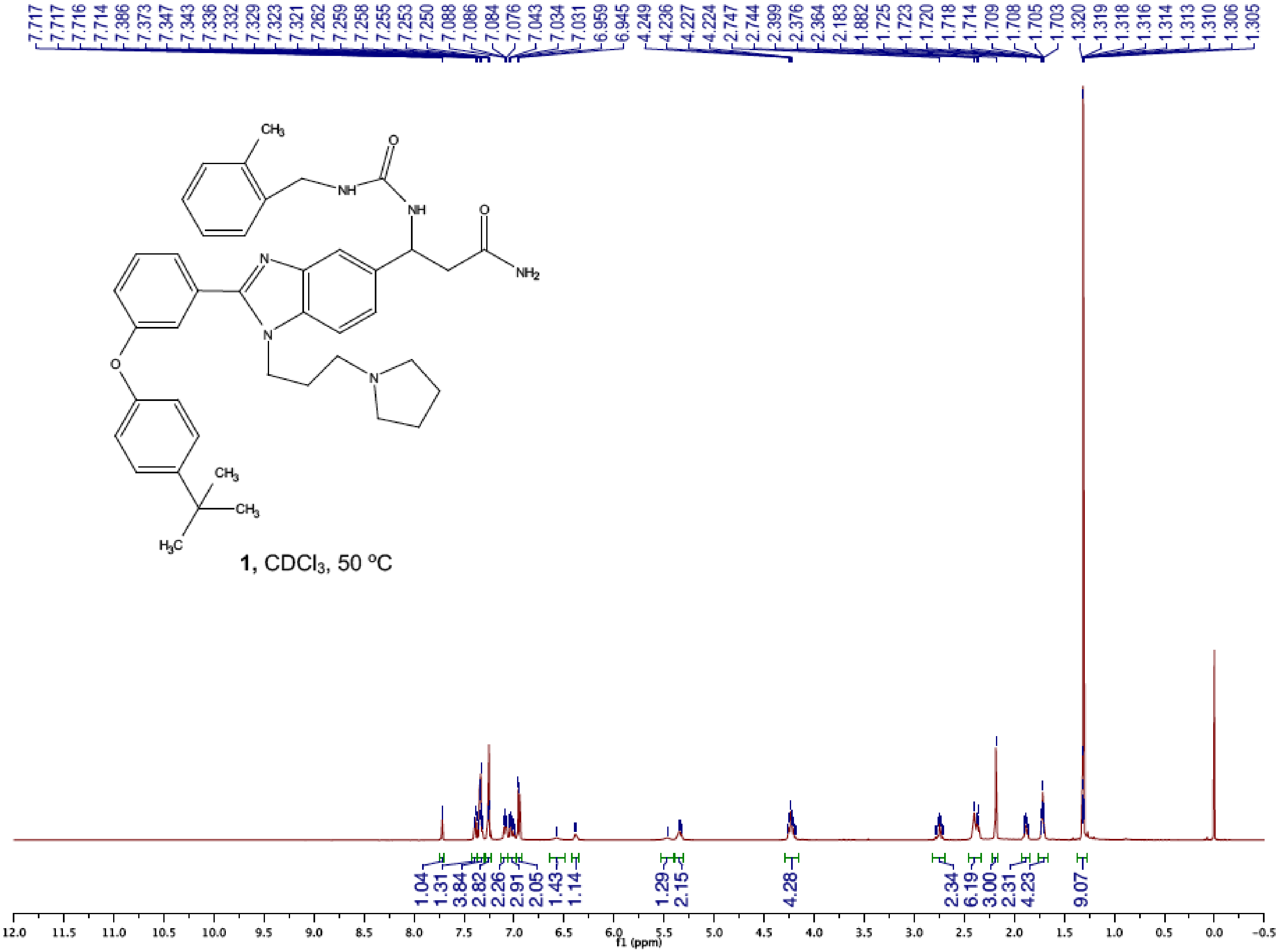

1, $CDCl_3$, 50 °C

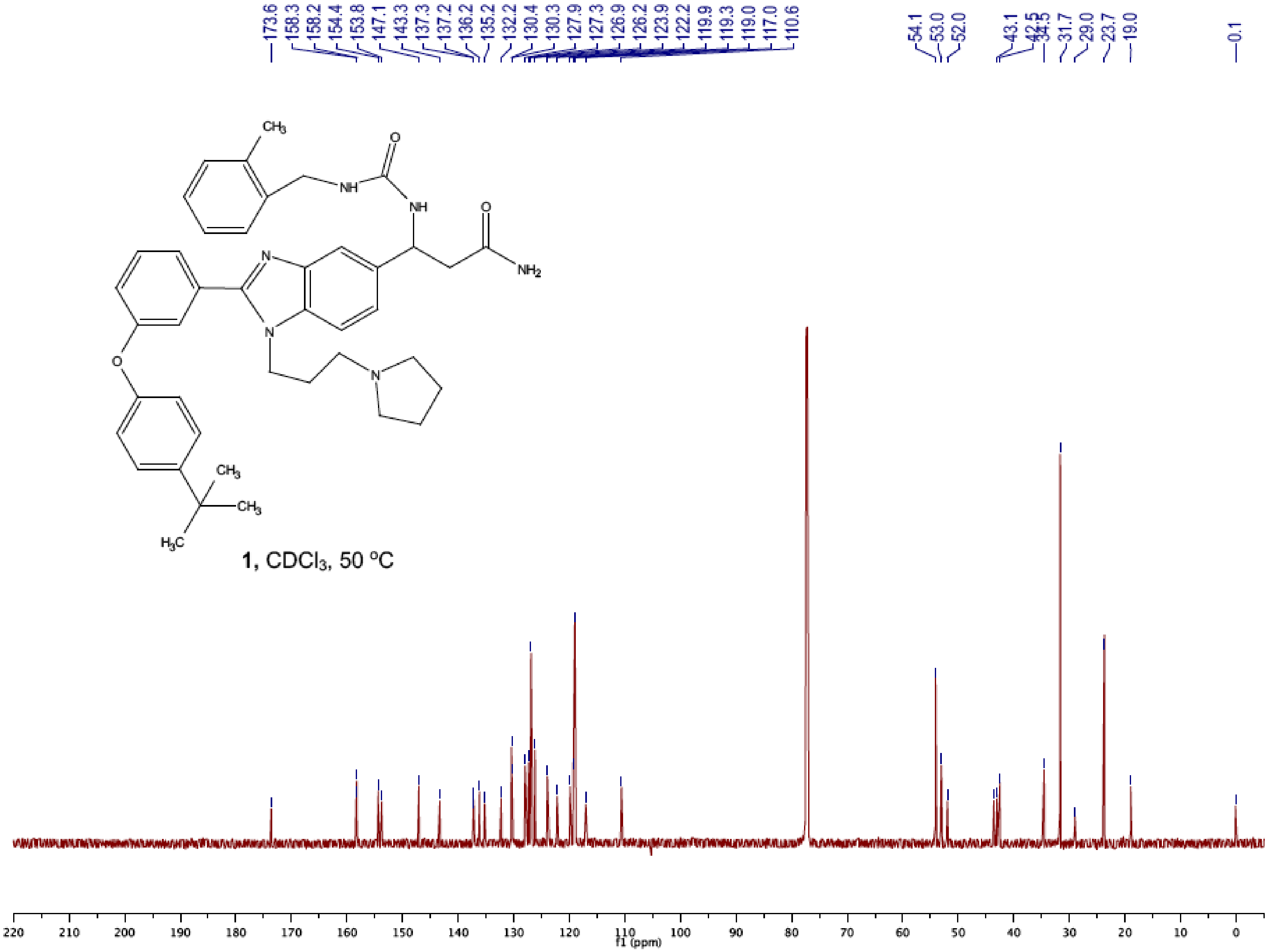
173.6
158.3
158.2
154.4
153.8
147.1
143.3
137.3
137.2
136.2
135.2
132.2
130.4
130.3
127.9
127.3
126.9
126.2
123.9
122.2
119.9
119.3
119.0
117.0
110.6
54.1
53.0
52.0
43.1
42.5
34.5
31.7
29.0
23.7
19.0
0.1
CH3
O
NH
NH
O
NH2
N
N
N
O
CH3
CH3
H3C
1, CDCl3, 50 °C
f1 (ppm)
